# Matrix Metalloproteinase-8 as an Inflammatory and Prevention Biomarker in Periodontal and Peri-Implant Diseases

**DOI:** 10.1155/2018/7891323

**Published:** 2018-09-16

**Authors:** Ahmed Al-Majid, Saeed Alassiri, Nilminie Rathnayake, Taina Tervahartiala, Dirk-Rolf Gieselmann, Timo Sorsa

**Affiliations:** ^1^Clinic of Preventive Dentistry, Periodontology and Cariology, Center of Dental Medicine, University of Zurich, Zurich, Switzerland; ^2^Department of Oral and Maxillofacial Diseases, University of Helsinki and Helsinki University Hospital, Helsinki, Finland; ^3^Karolinska Institutet, Department of Dental Medicine, Division of Periodontology, Stockholm, Sweden; ^4^Institute of Molecular Diagnostics, Dentognostics GmbH, Solingen and Jena, Germany

## Abstract

Levels of and especially the degree of activation of matrix metalloproteinase (MMP-8) in oral fluids (i.e., saliva, mouth rinse, gingival crevicular fluid (GCF) and peri-implantitis sulcular fluid (PISF)) increase to pathologically elevated levels in the periodontal and peri-implant diseases. This study aimed at collecting and collating data from previously published studies and determining whether active MMP-8 (aMMP-8) could serve as a biomarker for the diagnosis and prevention of periodontal and peri-implant diseases. The literature search identified a total of 284 articles. Out of 284 articles, 61 articles were found to be relevant. Data obtained from the selected studies were combined, and it indicated that aMMP-8 in oral fluids exerts the strong potential to serve as a useful adjunctive diagnostic and preventive biotechnological tool in periodontal and peri-implant diseases. aMMP-8 can be used alone or in combination with other proinflammatory and/or microbiological biomarkers.

## 1. Introduction

Periodontitis and peri-implantitis, globally common infection-induced oral inflammatory disorders of teeth and dental implants supporting soft and hard tissue, i.e., periodontium and peri-implatium, involve destruction of both soft and hard tissues, as active periodontal and peri-implant degradation (APD). Periodontal/peri-implant tissues are mainly made up of type I collagen. The proteolytic enzyme mainly responsible for the active periodontal/peri-implant soft and hard tissue degeneration (APD) is matrix metalloproteinase (MMP-8), also known as collagenase-2 or neutrophil collagenase. MMP-8 is a member of the MMP family. Structurally related but genetically distinct MMPs are Ca^2+^- and Zn^2+^-dependent endopeptidases capable of degradation of almost all extracellular matrix and basement membrane protein components both in physiologic repair and pathologic destruction of tissues, such as a breakdown of extracellular matrix in embryonic development, wound healing, and tissue remodeling [1].

The MMP family is divided into six protease groups: collagenases (MMP-1, MMP-8, and MMP-13), gelatinases (MMP-2 and MMP-9), stromelysins (MMP-3, MMP-10, and MMP-11), matrilysins (MMP-7 and MMP-26), member-type MMPs (MMP-14, MMP-15, MMP-16, MMP-17, and MMP-12), and other nonclassified MMPs, given their auxiliary contrasts [2]. Among all of these groups, the collagenase group is of particular relevance in periodontal disease as it can efficiently cleave native collagen fibers I, II, and III. MMP-8 has been categorized under the interstitial collagenase subgroup of the MMP family. Activities of MMPs are inhibited and regulated by the endogenous or natural tissue inhibitors of tissue inhibitors of MMP (TIMPs) and *α*2-macroglobulin [3]. The imbalance between MMPs and TIMPs often results in irreversible periodontal and peri-implant destructive pathology involving irreversible APD [3–5].

Recently, an increased level of MMP-8, especially in activated/active form (aMMP-8), in oral fluids is associated with and reflects periodontal and peri-implant inflammation/diseases especially in clinical active phases [3, 6–8]. Periodontal and peri-implant degeneration (APD) is caused by interstitial collagenase MMP-8 and not by bacterial enzymes [9]. MMP-8 is released from neutrophils by selective degranulation triggered by potent periodontopathogenic bacteria and their virulence factors together with host-derived proinflammatory mediators [3, 7]. Gingival fibroblasts, when stimulated by proinflammatory mediators, such as interleukin (IL)-1*β* and tumor necrosis factor-*α*, can produce collagenolytic MMPs including MMP-8 [10]. The level of active, but not latent or total, collagenase-2/MMP-8 reflects, predicts, and is related to progressive periodontal and peri-implant disease activity [11]. Elevated levels of aMMP-8 in oral fluids (saliva, mouth rinse, gingival crevicular fluid (GCF), and peri-implant sulcular fluid (PISF)) were found to be associated with clinical periodontal parameters, i.e., probing pocket depth (PPD), bleeding on probing (BOP), and clinical attachment loss (CAL) [12]. The levels of aMMP-8 decrease after successful periodontal and peri-implant treatments [7, 13, 14].

A number of studies that have been performed utilize point-of-care (PoC)/chair-side analysis of elevated aMMP-8 in saliva/oral fluids [15–17]. A study comparing a PoC immunoflow tool with the standard gold laboratory-based one concluded that concentration of aMMP-8 in oral fluids is useful in distinguishing periodontal diseases from healthy subjects [15]. Lateral flow immunoassay of aMMP-8 has been shown to have high sensitivity for at least two sites with BOP and two sites with deepened periodontal pockets [18]. Sorsa et al. demonstrated that immunofluorometric assay (IFMA) and DentoAnalyzer-PoC-test could detect aMMP-8 from GCF samples, and these methods are comparable with the chair-side/PoC aMMP-8 dip-stick test [6]. The Amersham enzyme-linked immunosorbent assay (ELISA) for total MMP-8 immunoactivities was not in line with the PoC/chair-side immune tests, specific for aMMP-8 [6]. Few studies demonstrated the associations of various periodontal pathogens in oral fluids with the levels of aMMP-8 and suggested to use in combination with aMMP-8 with other proinflammatory and microbiological biomarkers that may potentially improve the diagnostic accuracy [6, 7]. The present review aimed at collecting and collating the data from published literature regarding the potential of aMMP-8 in saliva/oral fluids to be used as a biomarker and predictor for periodontal and peri-implant diseases [6, 7, 19, 20].

## 2. Materials and Methods

### 2.1. Study Identification

A literature search was performed in two electronic databases PubMed and Cochrane to identify related studies of the past 15 years. In addition to this, other relevant studies were identified by manual searching. Keyword used for study identification in all databases were “MMP-8 and periodontal inflammation,” “MMP-8 and peri-implantitis,” and “MMP-8 and low-dose doxycycline.” The synonyms such as MMP-8, collagenase-2, and neutrophil collagenase were also searched in combination with periodontitis. The electronic search was done from November 11, 2016, to July 30, 2018.

### 2.2. Study Selection

All identified studies were screened, and the selection process was done on the basis of inclusion and exclusion criteria.

#### 2.2.1. Inclusion Criteria

Inclusion criteria are as follows:

1. Randomized controlled trials
2. Observational studies
3. Review articles
4. Studies included low-dose doxycycline/sub-antimicrobial dose doxycycline (L/SDD) as an adjunctive drug for treatment of periodontal diseases

#### 2.2.2. Exclusion Criteria

Exclusion criteria are as follows:

1. Written in language other than English
2. Case reports
3. Thesis
4. Animals studies
5. Diagnosis of periodontal disease was not written
6. Experimental gingivitis

## 3. Results

### 3.1. Study Selection and Data Abstraction

The literature search identified a total of 284 articles. Out of 284 articles, data of 61 articles were selected. Data obtained from selected studies were combined and summarized in the present study (Table 1).

### 3.2. Sources of MMP-8 in the Oral Cavity

A major source of MMP-8 (neutrophil-type MMP-8) in humans are degranulating triggered neutrophils, but MMP-8 (mesenchymal cell-type MMP-8) is also *de novo* expressed and secreted in small amounts by non-PMN-lineage cells such as epithelial cells, smooth muscle cells, fibroblasts, macrophages, and endothelial cells [74–77]. Neutrophil collagenase/polymorphonuclear leukocyte- (PMN-) derived collagenase-2/MMP-8 differs from interstitial collagenases secreted by other cells in that it is synthesized only during the myelocyte stage of development of neutrophils in the bone marrow and stored as a latent enzyme, i.e., latent pro-MMP-8 (Mr 85 kDa) within the specific granules of PMN. Pro-MMP-8 is rapidly released from activated PMN undergoing selective subcellular granule degranulation and is then activated through the cysteine switch mechanism often, but not always, associated with selective *N*-terminal proteolysis to yield the active form of the enzyme (Mr 65 kDa) and activation fragments [3, 74–77].

The main source of oral salivary collagenase is PMNs that enter the oral cavity through gingival sulcus [11, 75]. It is evident from the fact that collagenase was only detected in whole oral saliva of subjects and not in secretions of major oral salivary glands. Furthermore, whole oral saliva collected from edentulous subjects did not show a significant amount of collagenase [75].

Oral fluid (GCF, PISF, mouth rinse, and saliva) collagenases exert similarity with PMN- or neutrophil-type collagenase-2 (MMP-8). It degrades type I and II collagens significantly faster than the type III collagen. Its molecular weight is 65–70 kDa, same as collagenase of the PMNs/neutrophils/MMP-8 and gingival sulcus [3, 7]. It is activated by gold thioglucose, which only activates PMN/neutrophil collagenase [3, 7, 75, 77].

### 3.3. Active and Latent Forms

Most of the oral salivary collagenase found in a healthy mouth is in the latent form, whereas in case of periodontal or/and peri-implant disease patient(s), it is in active or activated (aMMP-8) form together with activation fragments [3, 43, 75, 77]. Studies done by Gangbar et al. and Lee et al. [11, 76] demonstrated that oral fluid active collagenase, but not latent, is related and reflects to progressive clinical periodontal disease activity, i.e., loss attachment or APD. aMMP-8 in oral fluids precedes, predicts, is associated with, and reflects on on-going or future/developing progressive, often hidden and subclinical, periodontal and peri-implant disease activity, i.e., CAL, APD, and active peri-implant degeneration [3, 6, 7, 17, 76, 77]. Significant correlations have been found between aMMP-8 and progressing severity of periodontal and peri-implant diseases [3, 4, 6, 7, 26]. Successful periodontal and peri-implant treatment significantly reduces aMMP-8 levels in oral fluids [3, 6, 7, 17, 78, 79].

### 3.4. MMP-8 and Correlation with Periodontal Diseases

It has been documented in several studies that salivary and oral fluids at aMMP-8 levels are higher in subjects with localized and generalized periodontitis than in healthy controls but the levels reduced after nonsurgical periodontal therapy, i.e., scaling and root planning (SRP) [12, 39, 41, 43, 53]. Furthermore, aMMP-8, but not latent/total MMP-8, levels could differentiate between periodontitis and gingivitis as well [59]. A slight increase in MMP-8 levels could be observed in case of gingivitis, which shows a decrease after dental prophylaxis or secondary preventive interventions [57].

Nwhator et al. demonstrated that aMMP-8, measured by lateral flow chair-side/PoC immunoassay (PerioSafe®), is directly proportional to the oral hygiene status [18]. It shows a positive correlation with chronic periodontitis and BOP but only in the presence of two or more sites having the deepened PPD of not less than 5 mm; these aMMP-8 PoC findings indicate that such deepened sites are APD affected. The sensitivity of immunoassay for a single site affected by chronic periodontitis was found to be less [18]. Levels of aMMP-8 in oral fluids have been demonstrated to correlate with clinical periodontal parameters in particularly PPD, and it also reflects the effect of treatment [12, 18, 44, 47, 54, 62]. Levels of aMMP-8 are not only associated with clinical periodontal parameters status but also showed significant association with radiological parameters. aMMP-8 levels have been shown to differentiate subjects with a severe bone loss with those with a slight bone loss [53, 58]. Izadi Boroujeni et al. demonstrated a sensitivity of 87% and specificity of 60% of aMMP-8 in a PoC detection of generalized chronic periodontitis [62].

In children, sites with aggressive periodontitis show higher levels of MMP than adults with chronic periodontitis [33]. Baeza et al. reported in their study that aMMP-8 levels in chronic periodontitis were elevated [69]. When aMMP-8 levels were measured by ELISA, the cutoff point was identified as 13 ng/ml chronic periodontitis case [69].

In number of previous investigations, aMMP-8 levels have been reported to predict periodontal disease progression [3, 7, 76, 77]. aMMP-8 levels differentiate between subjects with stable and progressing periodontitis; these confirmatory findings have been repeatedly recorded by independent immune and catalytic activity assays specific for aMMP-8 [6, 8, 80, 81]. While predicting periodontal disease progression, highest sensitivity was noted with salivary/oral fluid aMMP-8, whereas GCF aMMP-8 showed high specificity [56, 59, 63, 71].

Leppilahti et al. established cutoff levels for smoking and nonsmoking periodontal patients to predict site-specific levels of treatment outcomes [56, 57, 59, 63]. The most optimal cutoff value among smokers was 0.045, whereas for nonsmokers, the calculated value was 0.085. These values can be helpful in longitudinal monitoring of the disease status during the maintenance period [56, 57, 59, 63].

### 3.5. Study Specimens

Oral fluids, such as mouth rinse, GCF, PISF, and saliva, have been used as specimens [3, 6, 7]. Mouth-rinse samples can be collected quickly, noninvasively, and the collection process is less time-consuming as compared to a collection of GCF and PISF. Mouth-rinse assay is useful for screening purposes mainly, but it does not provide exact information or identification/localization about the sites of clinically active disease. Whole saliva, variation in the salivary flow rate, use of antimicrobial medication, and smoking habits may have an impact on the results. GCF and PISF provide site-specific information, therefore useful in the personalized treatment plan of an individual [53]. Johnson et al. reported that when measured with lateral flow immunoassay, saliva showed 4.1 times higher concentration of MMP-8 in periodontal patients than periodontal healthy controls [15].

Correlation between aMMP-8 levels in serum and oral fluids have been tested in few studies [34, 41, 56, 71]. Noack et al. reported a significant correlation between aMMP-8 concentration in the serum and severity of periodontal disease. In addition, serum MMP-8 concentration was also found to show a positive correlation with a subgingival bacterial load [71]. Differing from findings of Noack et al. [71], others on serum concentration of MMP-8 failed to find any correlation with periodontal disease [34, 41]. These varying associations can also be affected by differences in the use of clinical indices utilized to assess periodontal health and disease as well as systemic assessments of patients and healthy controls. Additionally, various mediations may affect systemic and serum aMMP-8. [34, 41, 56, 71] Only one study reported that fibroblasts were used as a study specimen to evaluate its role in the pathophysiology of peri-implantitis. [34] When proinflammatory and matrix degrading responses of gingival and granulation tissue fibroblasts to an *in vitro* challenge to *Porphyromonas gingivalis* (*P. gingivalis*) were compared between subjects with healthy periodontium and patients with periodontitis and peri-implantitis lesion, MMP-8 expression was found higher in nonchallenged peri-implantitis fibroblasts than in fibroblasts from healthy periodontium. This indicates that the inflammatory response was more pronounced in fibroblasts from periodontitis and peri-implantitis than in fibroblasts from periodontally healthy individuals. These findings suggest that the exposure of prolonged inflammation, i.e., periodontal/peri-implant disease experience and burden, can affect and promote cells' ability to express MMP-8 [34].

Passoja et al. did not find any correlation between periodontal disease and serum MMP-8 levels [34]. A study performed by Özçaka et al. showed that the levels of MMP-8 in the serum of patients with chronic periodontitis did not significantly differ from periodontal healthy subjects [41]. Kinney et al. showed that serum levels of biomarkers did not play any significant role in the diagnosis of periodontitis [56].

### 3.6. Immunoassays Used to Detect aMMP-8 (IFMA, DentoAnalyzer, DentoELISA, ELISA as Neutrophil Collagenase-2 Immunoassays)

MMP-8 detected by the IFMA technique correlates more strongly with the periodontal and peri-implant status, and better diagnostic accuracy is found higher than that of ELISA [58, 59]. A possible reason is that ELISA mostly detects all forms of MMP-8 (total/latent MMP-8), whereas IFMA selectively identifies activated neutrophil and fibroblast-type isoforms of MMP-8, then particularly in the active form (aMMP-8) [6]. A study done by Leppilahti et al. shows that results of IFMA were comparable with DentoELISA but not with commercial Amersham ELISA; IFMA and DentoELISA utilize the same aMMP-8 antibody [6–8, 18, 24, 28, 42, 64, 70, 82] Total MMP-8 levels measured by the Amersham ELISA test did not correlate with values of periodontal parameters [6–8, 42, 83].

**Figure 1:**
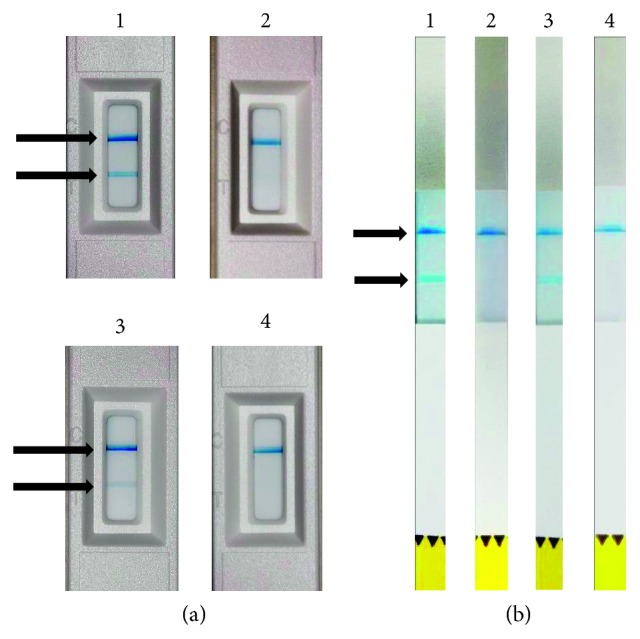
Periodontitis (a) results based on PerioSafe®-mouth-rinse test: two chronic periodontitis patients (1) and (3) before and (2) and (4) after nonsurgical periodontal treatment, scaling, and root planning (SRP). The appearance of two lines (>20 ng/ml) pointed by arrows in the figure is a considered positive test which indicates elevated risk for periodontitis. The appearance of only one line indicates successful test performance and no risk for periodontitis (aMMP-8 < 20 ng/ml) after SRP treatment. Peri-implantitis (b) results based on ImplantSafe®-PISF-strip-test; two peri-implantitis patients (1) and (3) before and (2) and (4) after peri-implantitis treatment (plastic scaling, oral hygiene instructions, and use of chlorhexidine). Two lines in the result window indicate elevated aMMP-8 in PISF and increased risk for peri-implantitis. The appearance of a single line indicates successful test performance, low aMMP-8 in PISF, and no risk for peri-implantitis after treatment [78, 84].

Baeza et al. reported aMMP-8, measured by IFMA, to be less accurate in differentiating periodontitis from healthy sites. Differing from the other studies, the performance of DentoELISA was comparable to IFMA [69]. In chronic periodontitis patients, a positive correlation was observed between PPD and aMMP-8, measured by IFMA. CAL showed a positive correlation with aMMP-8, measured by IFMA and DentoELISA.[69]. Lateral-flow chair-side/PoC-PerioSafe® and ImplantSafe® immunotests (Figure 1), with and without the quantitative reader ORALyzer®, utilized the same aMMP-8 antibody as IFMA and DentoELISA, and they all correlate well with each other [13, 17, 61, 65, 78, 84, 85].

### 3.7. aMMP-8 Level in Oral Fluids of Smokers

According to Mäntylä et al., the mean aMMP-8 levels in smokers were found to be lower compared to non-smokers, but sites with the progressive disease show similar or higher levels of aMMP-8 in both smokers and nonsmokers [28]. Heikkinen et al. found similar results when comparing levels of aMMP-8 levels between smokers and nonsmokers, but the difference found was not statistically significant. Levels of aMMP-8 reduced after SRP but sites with exceptionally elevated aMMP-8 concentrations clustered in smokers did not show a significant decrease in aMMP-8 after SRP. These sites with a poor response may indicate sites at elevated risk and were easily identified by the chair-side/PoC aMMP-8 test. [28, 38] Baseline GCF aMMP-8 levels have been shown to predict aMMP-8 levels during maintenance of periodontitis. Particularly in smokers, high levels of aMMP-8 at the baseline indicated a poor response to periodontal treatment [60].

According to Heikkinen et al., smoking affects the biomarker values in a dose-dependent manner. Former smokers were found to have a similar level of aMMP-8 as compared to nonsmokers. Furthermore, obesity was found to be a confounder. Values of aMMP-8 among nonsmokers did not remain statistically significant when body mass index values were taken into account during analysis. However, the values were not affected in case of male smokers [38].

In contrast to these studies, Passoja et al. and Miller et al. did not find any significant correlation of smoking with an elevated aMMP-8 level in their independent studies done on saliva and GCF, respectively [29, 34]. Results of a study by Gursoy et al. showed that aMMP-8 was higher in nonsmoking periodontitis patients than controls, and in smokers', only statistically significant parameter was TIMP-1 level that could differentiate between periodontitis patients and control. The ratio of aMMP-8, measured by the IFMA method, and TIMP-1 could successfully differentiate between periodontitis and healthy smoking subjects as well. A possible explanation for this finding, according to the authors, is that MMP-8 is less effective in mediating tissue degradation in the smoker subjects. It also indicates that smoking eventually can affect the detection of the potential biomarkers of periodontal disease [37].

### 3.8. MMP-8 Levels before and after Nonsurgical Therapy

Gonçalves et al. demonstrated that SRP and use of systemic antibiotics effectively reduced local levels of specific MMPs in case of localized aggressive periodontitis. [54] Leppilahti et al. showed in their study that in patients who underwent azithromycin antibiotic treatment, the MMP-8 levels in GCF specifically are more stable and remain lower than a predefined cutoff level [63].

A study done by Konopka et al. showed that SRP improves all examined clinical periodontal parameters, apart from CAL. However, the GCF levels of MMP-8 after therapy in the periodontitis patient was still found to be higher than a control group [47]. In contrast to this finding, Gonçalves et al., found that level of MMP-8 in GCF was comparable to healthy sites. Most marked reduction in MMP-8 levels was noticed in a short period, i.e., 3–6 months after receiving treatment [54].

Nonsurgical therapy with and without antibiotics can reduce the level of active and total collagenase/MMP-8 [11] At the beginning of the treatment, the total collagenase activity was found similar to that of active collagenase demonstrating that most of the collagenase present at this stage was in an active form [11]. However, Konopka et al. could not find any correlation between clinical parameters and amount of humoral factors after the therapy, while they showed a correlation at the baseline with PPD and a proximal plaque index (PI) [47]. Baseline GCF MMP-8 levels strongly predict the change in level during a maintenance period [59, 63]. Elevated baseline levels of GCF MMP-8 in smokers indicate a weak response to therapy [59, 60, 63].

### 3.9. Host Response Modulation

This term is recently introduced in dentistry and means modifying destructive aspects of inflammatory host response that develops in periodontal and peri-implant tissues as a result of inflammatory outcome to chronic subgingival bacterial plaque. The purpose of this therapy was to restore a balance between proinflammatory mediators and anti-inflammatory mediators. Host modulation by low-dose-doxycycline/sub-antimicrobial-dose-doxycycline (L/SDD) medication also efficiently inhibits and reduces gingival tissue and oral fluid aMMP-8 and at the same time ceases the progression of periodontal/peri-implant tissue destruction (APD) [3, 7, 66]. Only L/SDD has been licensed and accepted by FDA as a host response modulator and MMP-inhibitory drug in humans for the treatment of periodontal disease until now [66]. In L/SDD, doxycycline 20 mg is given orally twice a day or 40 mg once a day to produce serum levels of doxycycline, which is too low to produce any antimicrobial effects but enough effective to inhibit/downregulate aMMP-8 [7]. In contrast to traditional dose (100 mg, once, or twice daily), L/SDD does not cause any bacterial resistance to doxycycline and does not alter normal flora, a composition of bacterial biofilm and their susceptibility to doxycycline and other antibiotics, even after long-term (up to 24 months) daily administration [66]. Furthermore, L/SDD causes a significant reduction in the levels of inflammatory mediators, mediators of collagenolysis (= aMMP-8), collagen degradation products, proinflammatory cytokines, and periodontal connective tissue destruction. [32] It has been shown to inhibit alveolar bone loss during periodontitis due to its ability to reduce gingival oxidative stress and aMMP-8 [32].

Evidence suggests that L/SDD has a strong potential for modulation of host response in beneficially aiding disease management when used as an adjunct medication to conventional mechanical therapy, SRP [27]. L/SDD reduces postsurgical BOP, PPD, and periodontal bone resorption [66]. L/SDD has been shown to support periodontal treatment like SRP as well as reduce the related systemic low-grade inflammation [31].

Emingil et al. concluded in their study that use of L/SDD together with SRP in the chronic periodontitis patient showed better clinical results/treatment outcomes as compared to SRP alone. A significant decrease in gingival inflammation scores at 3 months, and PPD reduction at 9 months was observed in the L/SDD group compared to a placebo group and was maintained until the end of 12 months [25]. In a study, L/SDD caused 36% reduction of bone height loss, when added to periodontal maintenance [36]. Sorsa et al. concluded that L/SDD, when coupled with SRP, could inhibit the activity or decrease expression of host MMPs, especially aMMP-8, by a mechanism that is unrelated to its antimicrobial property [3, 6, 7].

### 3.10. Effect of Metal Restorations

According to a study done by Khuslinski et al. on practically healthy subjects with intact periodontium and patients with chronic generalized periodontitis with various structural materials of dental restorations [46], the level of MMP-8 surpassed the normal only in oral fluids of patients with chronic generalized periodontitis with metal restorations. In patients with chronic generalized periodontitis with or without metal dental restorations, obtained correlation coefficients indicate triggered biochemical cascade accompanied by the activation of cytokine production in response to etiological factors. The group of patients with periodontitis and metal restorations demonstrated a reaction that is more marked.

### 3.11. Association of Periodontal Microorganism with MMP-8

The presence of subgingival microorganisms, mainly *Treponema denticola (T. denticola)*, seemed to increase the levels of salivary albumin, the total protein contain in saliva, and levels of MMP-8 in GCF. There is a possibility that both *T. denticola* and *Tannerella forsythia* (*T. forsythia*) have induced a cascade-type host response with increased release and activation of MMP-8 in GCF [35, 50, 86]. *T. denticola* and *P. gingivalis*-derived proteases (dentosilin and gingipain, respectively) can proteolytically and efficiently activate and convert latent pro-MMP-8 to aMMP-8 [45, 86, 88].

### 3.12. MMP-8 and Genetic Background

According to Heikkinen et al., genetic polymorphism of MMP-3 and vitamin D receptor found to be linked to initial periodontitis in Finnish adolescents, and aMMP-8 PoC/chair-side immunoassay PerioSafe® mouth-rinse test can be used for on-line PoC detection of initial periodontitis or preperiodontitis in adolescent patients with such type of genetic predisposition. This indicates the preventive potential of the PerioSafe® ORALyzer®-aMMP-8 chair-side/PoC test [70]. Thus, aMMP-8 mouth-rinse chair-side/PoC test positivity and 3 or more >4 mm pockets associated with the vitamin-D receptor and MMP-3 single-nucleotide polymorphisms. No association was found between single nucleotide polymorphism studied with the positivity of aMMP-8 [70].

### 3.13. MMP-8 in Dental Peri-implantitis

An inflammatory reaction associated with loss of supporting bone beyond initial biological bone remodeling around a dental implant, called peri-implantitis, is commonly reported as one of the significant contributors to dental implant failure [88–91]. The etiopathogenesis in case of peri-implantitis shows considerable similarity to periodontitis and shows comparable bacterial colonization and exudate of immune cells [88]. Similar to the periodontitis, aMMP-8 levels were repeatedly found to be pathologically elevated in diseased PISF as well [21–23, 30, 35, 45, 68, 82]. Both PMN- and non-PMN-type MMP-8 isoforms particularly in active forms have been observed in PISF of peri-implantitis patients [21–23, 30, 82]. However, Wang et al. reported that MMP-8 alone was not able to differentiate peri-implantitis patients from healthy patients [67]. *T. denticola* and *Prevotella intermedia* were reported to show diagnostic ability in case of peri-implantitis [67, 72, 73]. Gingival inflammation showed correlation to aMMP-8 levels in PISF [22, 23]. Ma et al. found that aMMP-8 in PISF, assessed by IFMA, associated with enhanced bone loss indicating that aMMP-8 participated in peri-implant bone loss and osteolysis [21]. Ritzer et al. [49] demonstrated by the 24/7-chewing-gum MMP-8 assay that elevated levels of MMP-8 could be detected in peri-implantitis oral fluids confirming and further extending the findings of Teronon et al., Kivelä-Rajamäki et al., Xu et al., and Kivelä-Rajamäki et al. [21–23, 30, 82].

Ramseier et al. reported a positive correlation between MMP-8, PI, and BOP in both GCF and PISF [65]. Ziebolz et al. have demonstrated that PISF aMMP-8 levels can be kept successfully low during maintenance in patients undergoing successfully supportive implant therapy indicating that successful professional maintenance intervention is associated with low (<20 ng/ml) PISF aMMP-8 levels similar to the healthy peri-implant status [73, 78, 79]. Similar clinical parameters and MMP-8 levels were obtained with both zirconium and titanium abutments at the end of 1 year. However, initially, titanium abutment was reported to show higher PISF MMP-8 levels and probing depth [72].

Thus, elevated levels of aMMP-8 in PISF associate significantly and repeatedly with peri-implant inflammation and bone loss/osteolysis [21–23, 30, 78]. Low (<20 ng/ml) in aMMP-8 levels in PISF reflects and indicates healthy and/or successfully treated status peri-implantium (Figure 1) [73, 78, 79]. Pathologically elevated levels of aMMP-8 (>20 ng/ml) can be conveniently detected by a quantitative lateral flow aMMP-8 dip-stick test, i.e., ImplantSafe® (Figure 1) [17, 78].

### 3.14. Combining Other Biomarkers to Increase Diagnostic Accuracy

Simultaneous measurement of more than one oral fluid marker may allow more accurate prediction of periodontal inflammatory burden [53]. Combinations of the MMP-8 biomarker and pathogens that correspond with it (such as *T. denticola*) may give a more accurate prediction of periodontitis as compared to a single biomarker alone [35].

Gursoy et al. concluded in their study that proportional or combined use of oral salivary biomarkers increases diagnostic accuracy, particularly in smoker subjects [37]. It was found that the MMP-8/TIMP molar ratio and the combination of two biomarkers, MMP-8 and pyridinoline cross-linked carboxyterminal telopeptide of type I collagen (ICTP), were significantly higher in detecting periodontitis compared to MMP-8 test alone [37]. A study testing accuracy of the cumulative risk score calculated (CRS) from three salivary biomarkers (i.e., *P. gingivalis*, IL-1*β*, and MMP-8) was more accurate in the diagnosis of advanced periodontitis than any of the markers alone [40]. Leppilahti et al. suggested measurement of MMP-8 and TIMP-1 to obtain higher diagnostic accuracy [42]. A study performed by Rathnayake et al. proposed use of MMP-8/TIMP-1 molar ratio as markers of periodontal disease in a larger patient population [52]. Salminen et al. proposed combination of three biomarkers, i.e., MMP-8, IL-1*β*, and *P. gingivalis* (CRS) for diagnosis of periodontitis [54] The median concentration of these three was significantly higher in the moderate to severe periodontitis group as compared to controls. In addition, Ebersole et al. reported also that salivary levels of IL-1*β*, IL-6, and MMP-8 provide high diagnostic accuracy for periodontitis with high sensitivity and specificity [55]. Furthermore, MMP-8 levels were higher in patients diagnosed with chronic periodontitis and diabetic, but *P. gingivalis* did not affect much. Unlike MMP-8, *P. gingivalis* values remain unaffected in edentulous subjects. *P. gingivalis* successfully differentiated current smokers from former smokers and however, did not show correlation with BOP [54].

Therefore, using biomarkers and various pathogens in combination may improve accuracy in diagnosis; however, the complexity and costs to perform such tests routinely will increase considerably. Therefore, simpler, inexpensive, and readily available tests that have been shown to be sufficient alone to detect and quantify aMMP-8, such as PerioSafe® and ImplantSafe®/ORALyzer®, might be more desirable (Figure 1) [78].

### 3.15. PoC Tests

Chair-side and point-of-care (PoC) lateral flow immunotests for the detection of aMMP-8 in oral fluids are commercially available (i.e., PerioSafe® and ImplantSafe®) with the detection limit of 20 ng/ml (Figure 1). The tests resemble the pregnancy home test (Figure 1). The quantitative reader-equipped ORALyzer® PoC test of oral fluids that measure aMMP-8 is found useful in differentiating active and inactive periodontal and peri-implant sites and patients, predicting disease progression in future, and monitoring the responses to therapy during the maintenance phase [17, 78]. The benefits of using these aMMP-8 tests are that these can be used in clinical settings, are easy to use, are inexpensive, and give prompt quick results with high sensitivity and specificity (i.e., the sensitivity of 90% and specificity of 70–85%) [6, 7, 16, 17, 24, 28, 42, 44, 51, 62, 64, 70, 78].

Alassiri et al. demonstrated that quantitative, PerioSafe® and ImplantSafe® ORALyzer®, PoC/chair-side assays could conveniently diagnose and follow the treatment of periodontitis and peri-implantitis [17, 78]. Thus, these tests can detect subclinical, developing periodontitis and peri-implantitis and related collage degradation even before the appearance of clinical and radiographical signs [14, 16, 17, 73, 82]. These test alarm preperiodontitis and pre-peri-implantitis and identity preventively future periodontal and peri-implantitis breakdown. They thus make invisible destruction or onset of periodontal/peri-implant collagenolysis to be visible and detectable in an enough early and predictive manner allowing the identification and timing of the preventive interactions/treatment such as secondary prevention and/or supportive periodontal/peri-implant treatment [17, 61, 65, 73, 84, 85].

PerioSafe® and ImplantSafe® with digital readers are modern *in vitro* fast immunological diagnostic and prevention professional technologies/tests for examination of the oral/periodontal/peri-implant status of teeth and dental implants at different time intervals (at least once annually) to detect risk of silent or hidden periodontal, peri-implant tissue degeneration and alveolar bone loss even before they can be detected clinically or radiographically [16, 17]. The PoC tests also help in time preventive treatment which is necessary for long-term success of implants, periodontal tissues, and patients. Another aspect of it is that this is also healthy and economical for the patients and society.

Regarding the chair-side/PoC-aMMP-8 lateral flow immune tests (Figure 1), the appearance of only one line indicates the negative result that reveals normal condition/a healthy status (<20 ng aMMP-8 per ml), and appearance of two lines indicates increased risk (>20 ng aMMP-8 per ml) (Figure 1) for periodontitis and/or peri-implantitis, either already existing or developing periodontitis and peri-implantitis, identified by PerioSafe and ImplantSafe, respectively (Figure 1). These PoC tests can be used with the reader for quantitative analysis [17, 78].

For quantitative analysis, dip-stick tests should be placed in the corresponding compartment of the reader. Then, flap is closed, the compartment is pushed into ORALyzer®, and check mark is pressed. The ORALyzer® is designed in such a way that it automatically starts and measures the aMMP-8 levels after 5 min. Thus, the qualitative “eye”-estimated plus/minus test results are quantitatively expressed in ng/ml aMMP-8 PoC/chairside [17, 78].

## 4. Summary

The current review analyzed the potential of aMMP-8 as a potential diagnostic, predictive, and preventive adjunctive biomarker/biotechnological tool for periodontal and peri-implant diseases. The available evidence suggests that especially aMMP-8 in oral fluids reflect, associate, and predict well with the clinical periodontal parameters and outcomes as well as clinical disease activity of periodontitis and peri-implantitis together with evaluation of treatment outcomes [18, 44, 47, 54]. Only few studies failed to find the correlation between clinical CAL [47], and few others reported that MMP-8 levels are not correlated with BOP [62]. In addition, aMMP-8 levels were reported to be associated with radiological parameters too [53]. Importantly when evaluating these studies, it should be kept in mind that active/activated MMP-8, not MMP-8 or total latent MMP-8, is a biomarker of active and progressive periodontal and peri-implant disease [3, 4, 6–9, 11, 17, 76, 77, 80, 81].

Thus, pathologically and repeatedly elevated of aMMP-8 levels in saliva show the highest sensitivity and in GCF/PISF, the highest specificity [56]. aMMP-8 levels of mouth rinse and oral saliva can be useful for screening, whereas GCF/PISF levels could predict at site-specific level treatment outcomes and may be a useful adjunct in an individual/personalized treatment and monitoring plans. Thus, the aMMP-8 tests represent tools for personalized medicine [56]. aMMP-8 levels reduce after nonsurgical therapy, such as SRP. Most of the studies confirmed the effect of smoking on MMP-8 level, except few [29, 34]. Combination of other biomarkers (TIMP-1, IL-6, and IL-1*β*) and periodontal pathogens (such as *T. denticola* and *P. gingivalis*) with aMMP-8 in the detection of periodontal inflammation may increase accuracy, but aMMP-8 alone functions quantitatively very well [6, 7, 16, 17, 78]. Both IFMA and DentoELISA were found to be able to differentiate periodontitis from healthy subjects, but in general, IFMA was more accurate [6, 7, 16, 17]. Results obtained from Amersham ELISA were not in line with IFMA and DentoELISA. Lateral flow chair-side/PoC aMMP-8 immunoassay correlated well with clinical parameters of periodontitis but with at least two sites and extended better accuracy than BOP [18, 78]. Notably, invasive aMMP-8 PoC-tests cause always bacteremia, but noninvasive aMMP-8 PoC-tests never [78].

Despite their high sensitivity and specificity, aMMP-8 PoC-assays should be mainly used as adjunct tools to the clinical examination; mouth-rinse/salivary assays are useful for screening and dip-stick for site-specific personlized medical approaches. High levels of oral fluid MMP-8 in subjects with clinically “appearing” healthy periodontium/peri-implantium indicate silent “hidden,” developing future preperiodontitis and pre-peri-implantitis indicating early preventive supportive periodontal and peri-implant treatment [16]. In the case, oral fluid aMMP-8 is not treated to be <20 ng/ml, and these preperiodontitis and pre-peri-implantitis phases will often develop to be periodontitis and peri-implantitis with on-going collagenolytic APD. Elevated oral fluid aMMP-8 thus predicts, reflects, and precedes future APD, i.e., CAL of the teeth and dental implants [3, 7, 17, 76]. Thus, aMMP-8 PoC/chair-side tests make invisible hidden inflammation visible [17, 78].

Our literature review results are in line with previous studies. A review done by Sorsa et al. concluded that MMP-8 is a promising candidate for diagnosis and determination of progressive periodontitis and peri-implantitis and monitoring response to therapy and further extend them also to peri-implantitis and provides diagnostic tests to monitor follow treatment and adjunctive medication such as L/SDD [7].

A systematic review and a recent study were done by de Morais et al. and Alassiri et al. and they concluded the same and recommended use of MMP-8 as a quantitative biomarker of periodontal and peri-implant diseases adjunctive to clinical examination [17, 78, 92].

### 4.1. Clinical Implications

Repeatedly pathologically elevated levels of aMMP-8, but not total/latent MMP-8, in oral fluids (mouth rinse, saliva, GCF, and PISF) show the positive correlation with the clinical and radiological parameters of periodontitis and peri-implantitis. Oral fluid aMMP-8 levels reduce after periodontal therapy, i.e., SRP combined with host modulation and/or antimicrobial medication [7, 93]. Continuously and sustainably pathologically elevated oral fluid MMP-8 levels indicate and predict sites and patients with compromised disease outcomes regarding course, treatment, and maintenance. Importantly, the oral fluid PoC/chair-side tests can be utilized to predict time preventive interventions before the development of irreversible tissue destruction (APD) in periodontium and peri-implantium by indentifying and alarming the preperiodontitis and pre-peri-implantitis [17, 70].

### 4.2. Limitations of the Study

1. Only the articles written in English language were selected.
2. A literature search was performed in two databases, and additional articles have been chosen by manual searching, but it is possible that some relevant data are left behind.

### 4.3. Recommendations for Future Research

Further studies, especially longitudinal and perspective ones, should be conducted to explore the relationship between aMMP-8 with other biomarkers. Some factors such as obesity and gender reported as confounding factors should also be addressed more in detail. The relationship between serum concentration of aMMP-8 and periodontitis and peri-implantitis is still not clear and needs further investigations.

## Figures and Tables

**Table 1:** Summary of studies related to periodontitis, peri-implantitis, and L/SDD and level of MMP-8 in oral fluids.

	Study title and reference	Reference (year)	Objective	Sample source	Smoker	Form of detected MMP-8 and other markers	Study population	Diagnosis	Result
1	Collagenases in different categories of peri-implant vertical bone loss [21]	Ma et al. (2000) [21]	To investigate if level of collagenase-2 and collagenase-3 in PISF act as mediators in the process of bone destruction in peri-implantitis	PISF samples	N/A	aMMP-8 by IFMA	13 subjects aged from 23 to 89 years old	Peri-implantitis	Gingival Index is not a clinically important marker for bone loss, but aMMP-8 and MMP-13 in PISF are. They might participate in peri-implant osteolysis

2	Levels and molecular forms of MMP-7 (matrilysin-1) and MMP-8 (collagenase-2) in diseased human peri-implant sulcular fluid [22]	Kivelä-Rajamäki et al. (2003) [22]	To identify various isoforms of MMP-8 in PISF and its relationship with MMP-7	PISF samples	N/A	aMMP-8 levels were determined by the western immunoblot method with polyclonal anti-human-MMP-8	13 subjects aged from 21 to 86 years old	Peri-implantitis	The elevated levels of aMMP-8 and MMP-7 were identified in active forms in diseased PISF, but MMP-7 was less prominent. MMP inhibitors, potential future tissue protective drugs, seemingly do not interfere with the defensive antibacterial action of MMP-7 but can inhibit aMMP-8

3	Laminin-5 gamma2-chain and collagenase-2 (MMP-8) in human peri-implant sulcular fluid [23]	Kivelä-Rajamäki et al. (2003) [23]	To investigate the forms and concentration of MMP-8 and laminin-5 gamma2-chain in PISF and to find correlation of these two with clinical parameters (i.e., the recorded gingival and bone resorption) of peri-implantitis	PISF samples	N/A	aMMP-8 levels were determined by western immunoblot	13 subjects aged from 21 to 86 years old	Peri-implantitis	aMMP-8 is a important biomarker of peri-implantitis, but longitudinal studies are required to assess their use, either alone or in combination as molecular biochemical PISF markers, to predict the risk of progression of peri-implantitis, as well as to monitor the impact of treatment of the disease

4	Gingival crevicular fluid collagenase-2 (MMP-8) test stick for chair-side monitoring of periodontitis [24]	Mäntylä et al. (2003) [24]	To develop a test stick for detection of MMP-8 in GCF, to evaluate its diagnostic potential as point-of-care/chair-side test, and to monitor the response to treatment of periodontitis	GCF samples	N/A	aMMP-8 levels were determined by IFMA, and chair-side dip-stick was performed	29 subjects, age not applicable	Healthy, gingivitis, and chronic periodontitis	aMMP-8 GCF levels and chair-side test differentiated periodontitis from gingivitis, and healthy control sites. Scaling and root planing could be followed successfully by both PoC-/chair-side and IFMA

5	The effect of adjunctive low-dose doxycycline therapy on clinical parameters and GCF MMP-8 levels in chronic periodontitis [25]	Emingil et al. (2004) [25]	To compare effectiveness of LDD combined with nonsurgical periodontal therapy alone in reducing levels of MMP-8 in GCF and improving clinical parameters in patients with chronic periodontitis	GCF	12 nonsmokers. none of the subjects was a heavy smoker (i.e., not more than 10 cigarettes/day)	aMMP-8 levels determined by the immunofluorometric assay	30 subjects, 37 to 61 years of age	Chronic periodontitis	Randomized, double blind, placebo-controlled, parallel arm study. LDD improved the effects of nonsurgical periodontal therapy

6	Longitudinal analysis of metalloproteinases, tissue inhibitors of metalloproteinases and clinical parameters in GCF from periodontitis-affected patients [26]	Pozo et al. (2005) [26]	Assessment of periodontal disease performed through measurement of extracellular MMP-8, MMP-9, and their TIMP-1 and TIMP-2 in GCF	GCF samples	N/A	aMMP-8 levels were determined by immune-western blotting (Cat. MAB 3316, Chemicon International, Temecula, CA, USA), MMP-9 by zymography, and dot blot of TIMP-1 and TIMP-2 (Cat. sc-6832 and sc-6835, respectively, Santa Cruz Biotechnology, Santa Cruz, CA, USA)	24 subjects, 30 to 35 years old	Healthy, and chronic periodontitis	A different pattern of aMMP-8 in control and patient site was found. The study has established the significant correlation between the severity of periodontal disease and the actual aMMP-8. aMMP-8 and the low level of both TIMP-1 and TIMP-2 were found

7	Is the excessive inhibition of matrix metalloproteinases (MMPs) by potent synthetic MMP inhibitors (MMPIs) desirable in periodontitis and other inflammatory diseases? That is: “Leaky” MMPIs vs excessively efficient drugs [27]	Sorsa and Golub (2005) [27]	Comparison between SDD and tetracycline (non-antibacterial composition) with more potent MMP inhibitors	N/A	N/A	N/A	N/A	N/A	Letter to editor: beneficial clinical efficiency observed only with LDD

8	Monitoring periodontal disease status in smokers and nonsmokers using a gingival crevicular fluid matrix metalloproteinase-8-specific chair-side test [28]	Mäntylä et al. (2006) [28]	To evaluate the efficacy of the aMMP-8-specific chair-side dip-stick test in longitudinally monitoring the periodontal status of smoking and nonsmoking patients with chronic periodontitis, using aMMP-8 concentration in GCF	GCF samples	11 smokers and 5 nonsmokers were included in the study	aMMP-8 levels were determined by chair-side lateral-flow immunotests and IFMA	16 subjects, age not applicable	chronic periodontitis	Persistently elevated GCF aMMP-8 concentration were identified, and they indicated sites at enhanced risk; patients with inadequate response to conventional treatment were identified by PoC/chair-side test and IFMA

9	Matrix metalloproteinases: contribution to pathogenesis, diagnosis and treatment of periodontal inflammation [3]	Sorsa et al. (2006) [3]	To understand the role of MMPs and their inhibitors in pathogenesis, diagnosis, and treatment of periodontal inflammation	N/A	N/A	N/A	N/A	N/A	Review: beneficial LDD adjunctive medical can be monitored/followed by aMMP-8 PoC/chair-side test

10	Salivary biomarkers of existing periodontal disease: a cross-sectional study [29]	Miller et al. (2006) [29]	To determine the correlation between salivary biomarkers specific for periodontal tissue inflammation, collagen degradation, bone turnover, and clinical features of periodontitis	Unstimulated whole expectorated saliva samples	33.3 case subjects and 27.6 control subject smokers were included in the study	Total MMP-8 levels were determined by the ELISA kit (Quantikine, R&D Systems, minneapolis, MN, USA)	57 subjects, 28 to 61 years of age	Healthy, and chronic periodontitis	A salivary level of MMP-8 appears to serve as biomarker of periodontitis

11	Characteristics of collagenase-2 from gingival crevicular fluid and peri-implant sulcular fluid in periodontitis and peri-implantitis patients: pilot study [30]	Xu et al. (2008) [30]	To identify the difference in collagenolytic activity between healthy subjects and subjects with peri-implantitis and to find the correlation between severity of peri-implantitis and collagenase activity	GCF and PISF samples	Nonsmokers were included in the study	Both aMMP-8 and total MMP-8 levels were determined by western blot and DNP-octapeptide assay	29 subjects, 4 healthy, 5 gingivitis patients, 10 chronic periodontitis patients, 5 implants patients, 5 peri-implantitis patients, the age range 23 to 72 years old	Healthy, gingivitis, chronic periodontitis and peri-implantitis	Peri-implantitis PISF contained higher active aMMP-8 levels and activity than GCF from similar deep chronic periodontitis sites. GCF and PISF from severe chronic periodontitis and peri-implantitis exhibited the highest aMMP-8 from PMNs and fibroblasts

12	Host-response therapeutics for periodontal diseases [31]	Giannobile (2008) [31]	To study factors affecting hard and soft tissue degradation around the teeth and dental implants.	N/A	N/A	N/A	N/A	N/A	Review: SSD is a useful/beneficial adjunctive medication in periodontitis

13	Host response modulation in periodontics [32]	Preshaw (2008) [32]	To study the role of SDD in modulation of host response in periodontal disease management	N/A	N/A	N/A	N/A	N/A	Review: MMP-8 is a potential biomarker at periodontitis and LDD is a useful adjunctive medication

14	Matrix metalloproteinase levels in children with aggressive periodontitis [33]	Alfant et al. (2008) [33]	To figure out the MMP-1, -2, -3, -8, -9, -12, and -13 levels in a cohort of African American children with and without aggressive periodontitis	GCF samples	17 nonsmokers were included in the study	Total MMP-1, -2, -3, -8, -9, -12, and -13 levels were determined by the ELISA kit (SenzoLyte 520, AnaSpec, San Jose, CA, USA)	44 subjects with AgP, 7 to 19 years of age, and 12 healthy controls. 17 adults with chronic periodontitis 35 to 65 years of age	Healthy, chronic periodontitis, and aggressive periodontitis	MMP-8 levels were elevated in AgP sites relative to nondiseased sites in the same subjects, in siblings and controls and subjects with chronic periodontitis. MMPs associated with the AgP sites in children were generally elevated compared to an adult cohort with a history of chronic periodontitis

15	Matrix metalloproteinase-8 concentration in shallow crevices associated with the extent of periodontal disease [34]	Passoja et al. (2008) [34]	To study association between MMP-8 levels in shallow, gingival crevices and the extent of periodontal disease	GCF samples	20 nonsmokers and 28 smokers were included in the study	Total MMP-8 levels were determined by the ELISA kit (Quantikine, R&D Systems, Minneapolis, MN, USA)	48 subjects, 22 to 75 years old	Chronic periodontitis	Statistically significant association between MMP-8 concentration from shallow crevices and the extent of attachment level (AL) ≥ 4 mm (*p*=0.028) and AL ≥ 6 mm (*p*=0.001), in subjects with moderate to high plaque scores

16	Identification of pathogen and host-response markers correlated with periodontal disease [35]	Ramseier et al. (2009) [35]	To find out the ability of putative host and microbially derived biomarkers to identify periodontal disease status from whole saliva and plaque biofilm	Unstimulated whole saliva samples	0% healthy, 19% gingivitis, 36% mild chronic periodontitis, and 81% severe chronic periodontitis smokers were included in the study	Total MMP-8 and -9, calprotectin, and OPG levels were determined by the ELISA kit (Quantikine, R&D Systems, Minneapolis, MN, USA). *A.actinomycetemcomitans, C. rectus*, *F. nucleatum*, *P. intermedia*, *P. gingivalis*, *T. forsythia*, *and T. denticola* with a quantitative PCR assay, IL-1*β*, -2, -4, -5, -6, -10, and -13, TNF-*α*, (FN-*γ* by protein microarray (Whatman, Florham Park, NJ), and ICTP by radioimmunoassay (Immunodiagnostic Systems, Fountain Hills, AZ)	100 subjects, aged ≥ 18 years old	Healthy, gingivitis, and chronic periodontitis	Multiple combinations of biomarkers especially MMP-8, 9, and osteoprotegerin combined with red complex bacteria provided highly accurate predictions of periodontal diseases.

17	Association of GCF biomarkers during periodontal maintenance with subsequent progressive periodontitis [36]	Reinhardt et al. (2009) [36]	To find correlation between GCF biomarkers of inflammation and bone resorption and loss of periodontal attachment and bone	GCF	N/A	Total MMP-8 level was determined by the ELISA kit (Biosource, Camarillo, CA)	128 osteopenic postmenopausal females (not taking estrogen) 45 to 70 years of age	Good general health, from healthy and chronic periodontitis patients'	Placebo-controlled clinical trial: SDD targets elevated aMMP-8 with beneficial clinical outcome

18	Oral salivary MMP-8, TIMP-1, and ICTP as markers of advanced periodontitis [37]	Gursoy et al. (2010) [37]	To detect potential markers of advanced periodontitis in saliva. In addition, we compared two MMP-8 detection methods using IFMA and ELISA to differentiate periodontitis subjects from controls	Stimulated whole saliva samples	17.2% healthy and 52.3% chronic periodontitis smokers were included in the study	aMMP-8 levels were determined by IFMA, total MMP-8, MMP-14, and TIMP-1 levels were determined by the ELISA kit (Amersham, GE Healthcare, Buckingamshire, UK) and ICTP levels were measured by enzyme immunoassay (Orion Diagnostica Oy, Espoo, Finland)	165 subjects, aged ≥ 30 years old	Healthy and chronic periodontitis	Salivary aMMP-8, when used in combination with TIMP-1 and ICTP is a potential biomarker in the detection of advanced periodontitis. The detection of total MMP-8 by the traditional ELISA method technique is less accurate than the aMMP-8 IFMA technique

19	Associations between matrix metalloproteinase-8 and -14 and myeloperoxidase in gingival crevicular fluid from subjects with progressive chronic periodontitis: a longitudinal study [13]	Hernández et al. (2010) [13]	To associate the levels, molecular forms, isoenzyme distribution, and degree of activation of MMP-8 and MMP-14, MPO, and TIMP-1 in GCF from patients with progressive periodontitis at the baseline and after periodontal therapy	GCF samples	N/A	aMMP-8 levels were determined by western blot and IFMA. MPO levels were determined by the ELISA kit (Immundiagnostik, Bensheim, Germany). MMP-14 and TIMP-1 levels were determined by the ELISA kit (Biotrak, GE healthcare, amersham, Slough, UK)	25 subjects, 35 to 62 years old	Chronic periodontitis	High aMMP-8 and MPO levels and a high MPO/MMP-8 positive correlation were found in active and inactive sites at baseline. After treatment, decreases in MPO and aMMP-8 were seen, except for active sites in which MMP-8 differences were not significant

20	Smoking affects diagnostic oral salivary periodontal disease biomarker levels in adolescents [38]	Heikkinen et al. (2010) [38]	To investigate the association between salivary aMMP-8 and PMN elastase with commonly used periodontal health indices in a birth cohort of adolescents accounting for their smoking habits	Stimulated whole saliva samples	61 boys and 66 girls were smokers. 197 boys and 177 girls were nonsmokers	Active MMP-8 levels were determined by IFMA	501 subjects, 15 to 16 years old	Most subjects were chronic periodontitis	Smoking significantly decreased both biomarkers, including aMMP-8 studied

21	Detection of gingival crevicular fluid MMP-8 levels with different laboratory and chair-side methods [6]	Sorsa et al. (2010) [6]	To compare four methods for detection of MMP-8 in GCF	GCF samples	Smokers were included in the study, but exact number of smokers is not mentioned	aMMP-8 levels were determined by DentoAnalyzer (Dentognostics GmbH, Jena, Germany), IFMA, and chair-side lateral-flow immunotests (Medix Biochemica Ltd, Espoo, Finland).Total MMP-8 levels were determined by the ELISA kit (Amersham, GE healthcare, Buckingamshire, UK)	10 subjects, age not applicable	Healthy, gingivitis and chronic periodontitis	IFMA (aMMP-8) and DentoAnalyzer (aMMP-8) results can detect MMP-8 from GCF samples, and these methods are comparable. The chair-side dip-stick test (aMMP-8) results were well in line with these assays. The Amersham ELISA (total MMP-8) results were not in line with tests.

22	Gingival crevicular fluid levels of MMP-8, MMP-9, TIMP-2, and MPO decrease after periodontal therapy [39]	Marcaccini et al. (2010) [39]	To compare the levels of MMP-8, MMP-9, TIMP-1, TIMP-2, and MPO in GCF of chronic periodontitis patients and controls at the baseline and three months after nonsurgical therapy	GCF samples	N/A	Total MMP-8, MMP-9, TIMP-1, and TIMP-2 levels were determined by the ELISA kit (DuoSet R&D Systems, Inc., Minneapolis, MN, USA), and MPO levels were determined (Sigma chemical, Co., St. Louis, MO, USA)	42 subjects, 35 to 55 years old	Healthy, and chronic periodontitis	Level of all the markers except TIMP-1 was found to be higher in GCF of patients compared with controls. The elevated level decreased three months after periodontal therapy

23	Use of host-and bacteria-derived salivary markers in detection of periodontitis: a cumulative approach [40]	Gursoy et al. (2011) [40]	The salivary concentration of three different salivary markers *P. gingivalis*, IL-1*β*, and MMP-8 were calculated together to obtain the cumulative risk score for detection of periodontitis	Stimulated whole saliva samples	N/A	*P. gingivalis* with a quantitative real-time PCR assay, IL-1*β* levels were determined by the ELISA kit (Amersham), and aMMP-8 levels were determined by IFMA	165 subjects, aged ≥ 30 years old	Healthy, and chronic periodontitis	The results point to that a cumulative risk score, calculated from the three salivary biomarkers, detects periodontal status more accurately than any of the markers individually. However, it is still sufficient to distinguish the periodontitis patient from the healthy group. However, aMMP-8 is reliable when used alone

24	Smoking and matrix metalloproteinases, neutrophil elastase and myeloperoxidase in chronic periodontitis [41]	Özçaka et al. (2011) [41]	To investigate the possible relationship between smoking and serum concentration of aMMP-8, MMP-9, TIMP-1, MPO, and neutrophil lactase in chronic periodontitis patients relative to periodontally healthy subjects	Serum samples	Healthy subjects (17 smokers) and chronic periodontitis patients (16 smokers) were included in the study	aMMP-8 levels were determined by IFMA; MMP-9 levels were determined by Biotrak ELISA Systems, Amersham Biosciences Ltd, Buckinghamshire, UK; TIMP-1 levels were measured by Duoste ELISA Development Systems, R&D systems, MN, USA; MPO levels were measured by Immunodiagnostic AG, Bensheim, Germany; and neutrophil elastase by Bender MedSystems GmbH, Vienna, Austria	111 subjects, 33 to 65 years	Healthy, and chronic periodontitis	aMMP-8 concentration and aMMP-8/TIMP-1 molar ratios in chronic periodontitis group were not found to be significantly different from those in the periodontally healthy group

25	Oral rinse MMP-8 point-of-care immuno test identifies patients with strong periodontal inflammatory burden [42]	Leppilahti et al. (2011) [42]	To determine if MMP-8 (measured by three different methods), TIMP-1, and elastase activity differentiate subjects with the different periodontal conditions, and second, to find out if MMP-8 levels were comparable among the methods used	Oral-rinse samples	Smokers were included in study, but the exact number of smokers is not mentioned	aMMP-8 levels were determined by DentoELISA (Dentognostics GmbH, Jena, Germany) and IFMA; total MMP-8 levels were measured by the ELISA kit (Amersham, GE Healthcare, Buckingamshire, UK); TIMP-1 levels were determined by the ELISA kit (Amersham); and elastase activity by Sigma Co., St Louis, MO, USA	214 subjects, 44 to 78 years old	Chronic periodontitis	aMMP-8 testing of oral-rinse samples may be beneficial in periodontal diagnostics. Total MMP-8 levels were not useful in diagnosis

26	Salivary biomarkers of periodontal disease in response to treatment [43]	Sexton et al. (2011) [43]	To check utility of salivary biomarkers in the monitoring of periodontal disease over time in subjects who received localized periodontal therapy	Unstimulated whole saliva samples	23% of the SRP group, and 33% of the OHI group smokers were included in the study	Total MMP-8 and OPG levels were determined by the ELISA kit (Quantikine, R&D Systems, Minneapolis, MN, USA) and IL-1*β*, IL-8, MIP-1*α*, and TNF-*α* levels were measured by Luminex human cytokine/chemokine multiplex kits (Millipore, St. Charles, MO, USA)	68 subjects, aged ≥ 18 years old	Chronic periodontitis	Salivary levels of biomarkers, i.e., IL-1*β* MMP-8, OPG, and MIP-1*α* reflected disease severity and response to therapy suggesting their potential utility for monitoring periodontal disease status

27	Full-mouth profile of active MMP-8 in periodontitis patients [44]	Kraft-Neumärker et al. (2011) [44]	To investigate whether there was a relationship between clinical diagnostic parameters and the concentration of aMMP-8 in GCF in the site level full-mouth analysis	GCF samples	Nonsmokers were included in the study	aMMP-8 levels were determined by IFMA	9 subjects, 35 to 66 years old	Chronic periodontitis	A statistically significant relationship found between level of aMMP-8 and pocket depth

28	Matrix metalloproteinase-8 is the major potential collagenase in active peri-implantitis [45]	Arakawa et al. (2012) [45]	To compare levels of MMP-1, -8, and -13 in PISF of both healthy and diseased sites and to find correlation between these MMPs with bone loss	PISF samples	N/A	Total MMP-8, MMP-1, and MMP-13 levels were determined by Fuji Chemical Industry, Takaoka, Japan	64 subjects, the aged range 59 to 78 years old	Peri-implantitis	This study also showed MMP-8 as a possible marker for progressive bone loss in peri-implantitis

29	Matrix metalloproteinases and inflammatory cytokines in oral fluid of patients with chronic generalized periodontitis and various construction materials [46]	Kushlinskii et al. (2012) [46]	To compare oral fluid of practically healthy subjects with intact periodontium and patient with chronic generalized periodontitis with various structural materials of dental restorations	Oral fluid samples	N/A	Total MMP-8 levels were determined by the ELISA kit (Quantikine, R&D Systems, Minneapolis, MN, USA)	105 subjects, 18 to 52 years old	Chronic periodontitis	The MMP-8 level in oral fluid was found to be higher than the normal only in patients with chronic generalized periodontitis with metal restorations. No significant difference was found in the level of MMP-8 in patients of chronic generalized periodontitis without metal restoration

30	Effect of scaling and root planing on interleukin-1*β*, interleukin-8 and MMP-8 levels in gingival crevicular fluid from chronic periodontitis patients [47]	Konopka et al. (2012) [47]	To determine amounts of MMP-8, IL-8, and IL-1*β* in GCF from patients with chronic periodontitis in relation to clinical parameters	GCF samples	Nonsmokers were included in the study	Total MMP-8 and IL-8 and IL-1*β* levels were determined by the ELISA kit (Quantikine, R&D Systems, Minneapolis, MN, USA)	51 subjects, 30 patients (mean age 48.7 ± 9.1 years old), and 21 healthy subjects (mean age 33.7 ± 8.2 years)	Healthy, and chronic periodontitis	Short-term nonsurgical therapy resulted in significant improvement in periodontal indices and a marked decrease of MMP-8, IL-8, and IL-1*β* in GCF. However, the level of humoral factors was still higher than those in control group

31	Associations of periodontal microorganisms with oral salivary proteins and MMP-8 in gingival crevicular fluid [19]	Yakob et al. (2012) [19]	To investigate in subjects with and without periodontitis, the levels of salivary proteins and aMMP-8 in GCF in relation to the presence of specific periodontal pathogens	Unstimulated and stimulated whole saliva, and GCF samples	15 healthy, and 30 chronic periodontitis smokers were included in the study	aMMP-8 levels were determined by IFMA, *A.actinomycetemcomitans*, *P. intermedia*, *P. gingivalis T. forsythia*, *and T. denticola* with a quantitative PCR assay; Albumin was analyzed using an immunoturbidometric Tina-Quant® kit (Roche, Basel, Switzerland); the salivary immunoglobulin concentrations were then analyzed by ELISA [87]; and salivary total protein was measured using the colorimetric Lowry method [49]	101 subjects, mean age 59.2 ± SD 2.9	Healthy, and chronic periodontitis	Salivary albumin and protein concentration were significantly higher in subjects with *T. denticola.* Level of aMMP-8 was significantly higher in subjects with *T. denticola* and *T. forsythia*

32	Treponema denticola associates with increased levels of MMP-8 and MMP-9 in gingival crevicular fluid [50]	Yakob et al. (2013) [50]	To assess the association between the presence of site-specific subgingival microorganisms and the level of aMMP-8 and MMP-9 in GCF	GCF samples	15 healthy and 30 chronic periodontitis were included in the study	aMMP-8 levels were determined by IFMA, *A.actinomycetemcomitans*, *P. intermedia*, *P. gingivalis*, *T. forsythia*, and *T. denticola* with a quantitative PCR assay; MMP-9 levels were determined by the ELISA kit (Amersham, Biosciences UK Ltd, Buckinghamshire, UK)	99 subjects, mean age 59.2 ± 2.9	Healthy, and chronic periodontitis	The presence of *T.* forsythia and *T. denticola* was associated with increased levels of aMMP-8 in the test sites

33	Cytokine and matrix metalloproteinase expression in fibroblasts from peri-implantitis lesions in response to viable porphyromonas gingivalis [51]	Irshad et al. (2013) [51]	To analyze inflammatory reactions of fibroblasts after in vitro challenge with *P. gingivalis*	Fibroblasts	All subjects' nonsmokers were included in the study	Total MMP-8, -1 levels were determined by the ELISA kit (Quantikine Human, Pharmacia Biotech, Buckinghamshire, UK), TIMP-1 immunoassay (R&D Systems, Minneapolis, MN, USA), and *P. gingivalis* with a quantitative real-time PCR assay	Five patients periodontally healthy 54.4 ± (±18.7) years old, nine patients (II) 57.8 (±12.4) years old, seven peri-implantitis patients 54.4 (±9.2) years old	Peri-implantitis	Fibroblasts from peri-implantitis and periodontitis lesions gave a more pronounced inflammatory response to the *P. gingivalis* challenge than fibroblasts from healthy donors. They may therefore be involved in the development of inflammation in peri-implantitis and periodontitis. Moreover, the sustained upregulation of inflammatory mediators and MMP-1 in peri-implantitis fibroblasts may play a role in the pathogenesis of peri-implantitis

34	Salivary biomarkers of oral health: a cross-sectional study [52]	Rathnayake et al. (2013) [52]	Aimed to investigating if known salivary biomarkers could be used for epidemiological studies for detection of periodontitis	Stimulated whole saliva samples	75 smokers were included in the study	Active MMP-8 levels were determined by IFMA; TIMP-1 levels were measured by the ELISA kit; (Amersham); TNF-*α*, IL-1*β*, IL-6, and IL-8 were measured by Luminex Chemokine multiplex); lysozyme levels were measured the ELISA kit (Quantikine, R&D Systems, Minneapolis, MN, USA)	966 subjects, 20 to 89 years old	Chronic periodontitis	aMMP-8 could be used as marker of periodontal disease in more significant patient populations

35	Oral salivary type I collagen degradation end-products and related matrix metalloproteinases in periodontitis [53]	Gursoy et al. (2013) [53]	Type I collagen degradation end products and related MMPs were examined aiming at detecting potential markers of periodontitis in saliva with high sensitivity and specificity	Stimulated whole saliva samples	86 smokers were included in the study	Active MMP-8 levels were determined by IFMA; MMP-9 and MMP-13 levels were measured by the ELISA kit (Amersham, GE Healthcare, Buckinghamshire, UK); TRACP-5b levels were measured by BoneTRAP® assay, Immunodiagnostic Systems Ltd, Boldon, UK); ICTP levels were measured by enzyme immunoassay; (Orion Diagnostica UniQ ICTP, EIA; Orion Diagnostica, Espoo, Finland); CTx levels were measured by Serum CrossLaps® ELISA assay (Immunodiagnostic, Systems Ltd, Boldon, UK); and NTx levels were measured by OSTEOMARK® NTx; serum levels were measured by Wampole Laboratories (Princeton, NJ, USA)	230 subjects of ≥30 years old	Chronic periodontitis	aMMP-8 is a reliable biomarker candidate for detecting alveolar bone destruction

36	Periodontal treatment reduces matrix metalloproteinase levels in localized aggressive periodontitis [54]	Gonçalves et al. (2013) [54]	To evaluate MMP-1, -2, -3, -8, -9, -12 and -13 levels in the GCF after treatment of LAgP and to correlate these levels with clinical response	GCF samples	Nonsmokers were included in the study	Total MMPs levels were determined by the ELISA kit (SensoLyte 520, AnaSpec, Fremont, CA)	29 subjects of 5 to 21 years old	Aggressive periodontitis	Treatment of LAgP with Conventional mechanical treatment and systemic antibiotics reduced specific MMPs levels effectively. The significant association was observed between MMP-1, -2, -3, -8, -9, -12 and -13 and percentage of sites with PD > 4 mm

37	Patterns of salivary analytes provide diagnostic capacity for distinguishing chronic adult periodontitis from health [55]	Ebersole et al. (2013) [55]	To determine to analyze expression levels in unstimulated whole saliva samples collected from multiple occasions from 30 healthy adults and 50 chronic adult periodontitis patients	Unstimulated whole saliva samples	Only nonsmokers were included in study	Total MMP-8 levels were determined by the ELISA kit (Quantikine, R&D Systems, minneapolis, MN, USA)	80 subjects of 18 to 45 years old	Healthy and chronic periodontitis	Salivary levels of MMP-8 were significantly elevated in periodontitis patients compared with the daily variation observed in healthy adults

38	Clinical correlates of a lateral-flow immunoassay oral risk indicator [18]	Nwhator et al. (2014) [18]	To investigate the clinical correlates of a lateral-flow immunoassay with BOP, oral hygiene, and periodontal probing depth on the first time	Oral-rinse samples	5 smokers and 71 nonsmokers were included in the study	aMMP-8 levels were determined by chair-side lateral-flow immunotests (Dentognostics GmbH, Jena, Germany)	76 subjects, age not applicable	Healthy, and chronic periodontitis	The chair-side aMMP-8 immunoassay showed a high (82.6%) sensitivity for at least two sites with BOP and periodontal pockets. It showed a lower relationship with single-site periodontal pockets and BOP

39	Crevicular fluid biomarkers and periodontal disease progression [56]	Kinney et al. (2014) [56]	Assess the ability of a panel of GCF biomarkers as predictors of periodontal disease progression	Unstimulated whole saliva samples	0% was healthy, 19% were gingivitis, 36% were mild chronic periodontitis, and 81% were severe chronic periodontitis smokers were included in the study	Total MMP-8 levels were determined by the ELISA kit (Quantibody human cytokine array by RayBiotech, Inc., Norcross, GA, USA)	100 subjects, aged ≥ 18 years old	Healthy, gingivitis, and chronic periodontitis	MMP-8 was significantly higher in periodontal disease progression group compared to stable patients

40	Salivary biomarkers associated with gingivitis and response to therapy [57]	Syndergaard et al. (2014) [57]	The primary aim was to compare the concentrations of IL-1*β*, IL-6, PGE_2_, MMP-8, and MIP-1*α* in the whole saliva from patients with gingivitis with concentrations of these substrates in the saliva of patients with a clinically healthy periodontium	Unstimulated whole saliva samples	N/A	Total MMP-8, IL-1*β*, IL-6, PGE_2_, and MIP-1*α* levels were determined by ELISA kit, assay design, Ann Arbor, MI & EMD, millipore, Billerica, MA	80 subjects of 23 to 38 years old	Healthy and gingivitis	Concentrations of IL-1*β*, IL-6, and MMP-8 cannot distinguish gingivitis from health

41	Oral salivary biomarkers of bacterial burden, inflammatory response, and tissue destruction in periodontitis [58]	Salminen et al. (2014) [58]	To investigate the association of selected salivary biomarkers with periodontal parameters and validate the use of a novel salivary diagnostic approach, the cumulative risk score (CRS), in detection of periodontitis in subjects with angiographically verified coronary artery disease diagnosis	Stimulated whole saliva samples	58 were current smokers and 202 former smokers were included in the study	aMMP-8 levels were determined by IFMA, IL-1*β* was measured by flow cytometry-based Luminex kits, Milliplex, Map Kit; MPXHCYTO-60k, Millipore, Billerica, MA, USA, and *P. gingivalis* with a quantitative PCR assay was performed	493 subjects, age nonapplicable	Chronic periodontitis	The high salivary concentration of aMMP-8, IL-1*β*, and *P. gingivalis* was associated with deepened periodontal pockets and alveolar bone loss. aMMP-8 performed better compared to BOP%

42	Matrix metalloproteinases and myeloperoxidase in GCF provide site-specific diagnostic value for chronic periodontitis [59]	Leppilahti et al. (2014) [59]	To identify the diagnostic accuracy of GCF candidate biomarkers to discriminate periodontitis from inflamed and healthy sites and to compare the performance of two independent MMP-8 immunoassays	GCF samples	5 subjects healthy (nonsmokers), 3 nonsmokers with gingivitis, and 3 nonsmokers with chronic periodontitis were included in the study	aMMP-8 levels were determined by IFMA, and total MMP-8 was measured by ELISA kits, GE Healthcare, Amersham	25 subjects, healthy (mean age, 48.2 ± 11.2 years) gingivitis (mean age, 35.7 ± 15.4 years) and periodontitis patients (mean age, 46.0 ± 5.0 years)	Healthy, gingivitis, and chronic periodontitis	MMPs are highly discriminatory biomarkers for site-specific diagnosis of periodontitis. The comparison of two quantitative MMP-8 methods demonstrated IFMA to be more accurate than ELISA

43	Gingival crevicular fluid matrix metalloproteinase-8 levels predict treatment outcome among smokers with chronic periodontitis [60]	Leppilahti et al. (2014) [60]	To explore different GCF aMMP-8 patterns in smokers and nonsmokers with chronic periodontitis and test the utility of baseline GCF aMMP-8 levels in predicting categorically assessed treatment outcomes	GCF samples	10 smokers and 5 nonsmokers were included in the study	aMMP-8 levels were determined by IFMA	15 subjects, aged 28 to 64 years	Chronic periodontitis	Baseline aMMP-8 level in GCF strongly predicts how aMMP-8 levels behave during the maintenance period. In this regard, aMMP-8 analysis can be considered more useful than BOP. In smokers' sites, high baseline aMMP-8 levels indicate and predict weak treatment response

44	Targeted salivary biomarkers for discrimination of periodontal health and disease(s) [61]	Ebersole et al. (2015) [61]	Saliva-based diagnostic approach for periodontal health and disease based upon the abundance of salivary analyses coincidence with the disease	Unstimulated whole saliva samples	28 current smokers were included in the study	Total MMP-8 levels were determined by ELISA kit, the MILLIPLEX MAP Kit, EMD millipore, Billerica, MA, USA	209 subjects, aged ≥ 18 years	Healthy, gingivitis, and chronic periodontitis	Demonstrated the utility of MMP-8 in differentiating periodontitis from health

45	Activated matrix metalloproteinase-8 in saliva as diagnostic test for periodontal disease? a case-control study [62]	Izadi Borujeni et al. (2015) [62]	To evaluate sensitivity and specificity of a chair-side test for aMMP-8 to detect periodontitis	Oral-rinse samples	25 smokers were included in the study	aMMP-8 levels were determined by chair-side lateral-flow immunotests, Dentognostics GmbH, Jena, Germany	60 subjects, aged ≥ 18 years	Healthy and chronic periodontitis	Positive results of the aMMP-8 test significantly correlate with generalized chronic periodontitis. The test shows 87% sensitivity and 60% specificity in the diagnosis of chronic periodontitis

46	The utility of gingival crevicular fluid matrix metalloproteinase-8 response patterns in prediction of site-level clinical treatment outcome [63]	Leppilahti et al. (2015) [63]	To study different response patterns of MMP-8 among smoker and nonsmoker subjects with CP and GAgP to test its utility in predicting site level treatment outcome	GCF samples	86 smokers were included in the study	aMMP-8 levels were determined by IFMA	158 subjects, aged 27 to 49 years	Chronic periodontitis and aggresive periodontitis	Distinct types of MMP-8 response patterns were obtained for smokers and nonsmokers. Optimal cutoff levels of aMMP-8 defined for smokers and nonsmokers, which indicate risk for compromised treatment outcome at baseline and during maintenance

47	Pilot study on oral health status as assessed by an active matrix metalloproteinase-8 chair-side mouth rinse test in adolescents [64]	Heikkinen et al. (2016) [64]	To investigate whether a PoC mouth rinse test based on aMMP-8 immunoassay could identify patients with oral inflammatory burden among adolescents with early pathologic findings	Mouth rinse samples	5 smokers, and 42 nonsmokers were included in the study	aMMP-8 levels were determined by chair-side lateral-flow immunotests (Dentognostics GmbH, Jena, Germany)	47 subjects, aged 15 to 17 years	Chronic periodontitis	PoC/chairside was found to be useful in the online detection/diagnosis of oral inflammatory burden, i.e., periodontitis in adolescents with early, initial signs of periodontitis. Detection of caries is also possible but with less efficiency. The test shows 76.5% sensitivity and 96.7% specificity in the diagnosis of initial chronic periodontitis

48	Host-derived biomarkers at teeth and implants in partially edentulous patients. A 10-year retrospective study [65]	Ramseier et al. (2016) [65]	To compare host-derived biomarkers in PISF and in GCF from adjacent teeth and to analyze their level in both periodontal disease and healthy condition	PISF and GCF samples	Smokers were included in study but exact number of smokers is not mentioned	IL-1*β*, MMP-3, MMP-8, MMP-1, and MMP-1/TIMP-1 levels were determined by ELISA kits, R&D systems, Europe Ltd, Abingdon, UK	Total 997 samples were evaluated	chronic periodontitis and peri-implantitis	Increased levels of MMP-8 and IL-1*β* in PISF or GCF may be associated with inflammation around teeth and implants while lower levels of MMP-8/TIMP-1 may be an indicator of disease progression around implants and eased levels of MMP-8 and IL-1*β* in PISF or GCF may be associated with inflammation around teeth and implants while lower levels of MMP-1/TIMP-1 may be an indicator of disease progression around implants

49	Non-antibacterial tetracycline formulations: host-modulators in the treatment of periodontitis and relevant systemic diseases [66]	Golub et al. (2016) [66]	To address the evidences supporting adjunctive use of host modulation therapy with scaling and root planning in the long-term management of periodontal disease	N/A	N/A	N/A	N/A	N/A	Review: aMMP-8 PoC test is suitable to monitor the adjunctive beneficial SDD in periodontitis

50	Analysis of matrix metalloproteinases, especially MMP-8, in GCF, mouth rinse, and saliva for monitoring periodontal diseases [7]	Sorsa et al. (2016) [7]	To review recent studies related to monitoring of periodontal and peri-implant diseases by analyzing systemic and oral fluid biomarkers	N/A	N/A	N/A	N/A	N/A	Review: SDD targets increased aMMP-8 beneficial clinical outcome and no development bacterial resistance

51	Protein biomarkers and microbial profiles in peri-implantitis [67]	Wang et al. (2016) [67]	To assess diagnostic ability of biomarkers when combined with microbial profiles	PICF samples	4 current smokers and 21 past smokers were included in the study	Total MMP-8, OPG, IL-1*β*, TIMP-2, and vascular endothelial growth factor levels were determined by ELISA kits, custom human Quantibody, arrays, RayBiotech, Inc., Norcross, GA, USA, and *A.actinomycetemcomitans*, *P. intermedia*, *P. gingivalis, T. forsythia*, and *T. denticola* with a quantitative PCR assay	68 subjects, age range: 37 to 83 years	Peri-implantitis	The present data suggest that the increased levels of the selected PICF-derived biomarkers of periodontal tissue inflammation, matrix degradation/regulation, and alveolar bone turnover/resorption combined with site-specific microbial profiles may be associated with peri-implantitis and could have potential as predictors of peri-implant diseases

52	Peri-implant sulcus fluid (PISF) matrix metalloproteinase (MMP) -8 Levels in peri-implantitis [14]	Thierbach et al. (2016) [14]	To assess MMP-8 levels in PISF from diseased sites in both smokers and nonsmokers	PISF samples	17 smokers were included in the study	aMMP-8 levels were determined by DentoELISA immunoassay (Dentognostics, Jena, Germany)	29 subjects, 8 healthy patients, 3 gingivitis, and 18 chronic periodontitis	Peri-implantitis	aMMP-8 levels increase in peri-implantitis affected implants both in nonperiodontitis and periodontitis patients, but levels still after treatment of the condition reflect intensified host response around implants and indicate challenges of controlling peri-implantitis with any treatment modality

53	Correlation between peri-implant sulcular fluid rate and expression of collagenase2 (MMP8) [68]	Janska et al. (2016) [68]	To identify correlation between PISF and collagenase-2 level in superficial and fundus area of PI sulcus	PISF samples	N/A	aMMP-8 levels were determined by DentoELISA immunoassay (Dentognostics, Jena, Germany)	15 subjects, the age range 43 to 75 years	Peri-implantitis	Examination of aMMP-8 is a sensitive method when examining early inflammatory changes but depends from the depth of the sample collection in the gingival pocket

54	Rapid assessment of oral salivary MMP-8 and periodontal disease using lateral flow immunoassay [15]	Johnson et al. (2016) [15]	To determine the efficacy of a novel POCID for detecting MMP-8 concentration in oral fluids in comparison with a gold standard laboratory-based immunoassay	Unstimulated whole saliva samples	10 smokers were included in the study	Total MMP-8 levels were determined by rapidassays, ApS, Copenhagen-S, Denmark, EMD Millipore, Billerica, MA and luminex, Austin, TX, USA	41 subjects, aged 18 years or older	Healthy and chronic periodontitis	MMP-8 can be detected by POCID and concentration correlates with luminex for both saliva and rinse fluids. This study confirmed and further extended the original studies of Nwhator et al. [18] and Heikkinen et al. [64]

55	Diagnostic accuracy for apical and chronic periodontitis biomarkers in gingival crevicular fluid: An exploratory study [69]	Baeza et al. (2016) [69]	Assessment of level and diagnostic accuracy of an asset of potential biomarkers in GCF from patients with chronic periodontitis and AAP	GCF samples	19 smokers were included in the study	aMMP-8 levels were determined by IFMA, MPO levels were determined by ELISA kit, immunodiagnostik, AG, Bensheim, Germany. IL-1*β*, IL-6, TNF-*α*, Dkk-1, ON, PTN, TRAP-5, and OPG levels were determined by Multiplex detection panels Millipore, St. Charles, MO, USA, Magpix, Millipore, St. Charles, MO, USA, and MMP-2 and -9 levels were determined by gelatin zymography.	106 subjects, aged 44 to 52 years	Chronic periodontitis	aMMP-8 shows diagnostic potential for both chronic periodontitis and AAP. aMMP-8 was found to be higher in chronic periodontitis, followed by AAP

56	Pilot study on the genetic background of an active matrix metalloproteinase-8 test in finnish adolescents [70]	Heikkinen et al. (2017) [70]	To determine whether aMMP-8 chair-side test can detect initial periodontitis and caries with genetic background in adolescents	Oral fluid and DNA samples	5 smokers and 42 nonsmokers were included in the study	aMMP-8 levels were determined by chair-side lateral-flow immunotests (Dentognostics GmbH, Jena, Germany)	47 subjects aged 15 to 17 years	Chronic periodontitis	The aMMP-8 chair-side test has potential to detect initial periodontitis in adolescents with predisposing genetic background. aMMP-8 PoC/chair-side test acts as a gene test

57	Association of oral fluid MMP-8 with periodontitis in swiss adult subjects [12]	Mauramo et al. (2017) [12]	To find association between periodontitis and levels of aMMP-8 in saliva and GCF	Stimulated whole saliva and GCF	Never smokers were 150 (58.1%). Former smokers were 70 (27.1%). Current smokers were 38 (14.7%)	aMMP-8 levels were determined by IFMA	258 subjects, mean age 43.5 (21–58) years	Healthy and chronic periodontitis	Elevated levels of aMMP-8 in saliva and GCF are significantly associated with periodontitis in a systemically healthy adult

58	Association between serum and oral matrix metalloproteinase-8 levels and periodontal health status association between serum and oral matrix metalloproteinase-8 levels and periodontal health status association between serum and oral matrix metalloproteinase-8 levels and periodontal health status [71]	Noack et al. (2017) [71]	To identify the association between extent of circulating aMMP-8 and status of periodontal disease and aMMP-8 levels in oral fluids	Unstimulated whole saliva, stimulated whole saliva, GCF, and serum samples	Smokers were included in study but exact number of smokers is not mentioned	aMMP-8 levels were determined by IFMA, PerioSafe plus, (Dentognostics GmbH, Jena, Germany), and *A.actinomycetemcomitans, P. intermedia, P. gingivalis, T. forsythia,* and *T. denticola* with a semiquantitative PCR assay	59 subjects, aged 23 to 58 years	Healthy, gingivitis, and chronic periodontitis	The serum levels correlated significantly with oral aMMP-8 as well as with clinical periodontal parameters in a dose-dependent manner in systematically healthy subjects

59	Influence of different forms and materials (zirconia or titanium) of abutments in peri-implant soft-tissue healing using matrix metalloproteinase-8: a randomized pilot study [72]	Kumar et al. (2017) [72]	To compare peri-implant connective tissue response around titanium and zirconia abutments	PISF samples	Nonsmokers were included in the study	Total MMP-8 levels were determined by ELISA, Boster Biological Technology Co Ltd	12 subjects, the age range 20 to 45 years	Healthy	This study suggests the presence of more remodeling and/or inflammatory phenomena around titanium implant abutments than around zirconia abutments of a different design during the early stages but not at 1 year

60	Microbiological and aMMP-8 findings depending on peri-implant disease in patients undergoing supportive implant therapy [73]	Ziebolz et al. (2017) [73]	To study relation of microbiological findings and aMMP-8 level with peri-implant mucositis and peri-implantitis in subjects receiving periodontal or implant therapy	PISF samples	17 smokers with 43 implant sites	aMMP-8 levels were determined by DentoELISA (Dentognostics GmbH, Jena, Germany)	89 subjects with 171 implants. Mean age: 52 ± years, 116 dental implants were healthy, 39 dental implants had mucositis, and 16 dental implant had peri-implantitis	Peri-implantitis, peri-mucositis around implants, and chronic periodontitis	Within the limitations of this study, microbiological findings and aMMP-8 levels are not suitable for a differentiation between healthy, peri-mucositis, and peri-implantitis in patients all undergoing SIT/SPT. No healthy and disease patients without SIT/SPT were involved. Only smoking and the presence of Pi appear to be potential parameters associated with peri-implant disease in SIT/SPT patients. SIT/SPT intervention downregulated aMMP-8 during maintenance

61	Diagnosing peri-implant disease using the tongue as a 24/7 detector [49]	Ritzer et al. (2017) [49]	Anyone, anywhere, and anytime diagnostics were developed for peri-implant disease. The sensors responded to MMPs and provided proof of concept in statistically differentiating patients with peri-implant disease from healthy volunteers	Oral fluid	No-smokers were included in the study	aMMP-8 levels were determined by DentoELISA (Dentognostics GmbH, Jena, Germany) and MMP-8 analyzed by 24/7 chewing gum	33 subjects saliva or sulcus fluid collected from patients with peri-implant disease (defined as mucositis or peri-implantitis; *n*=19) and healthy control (*n*=14)	Peri-implantitis	Elevated MMP-8 could be detected in peri-implantitis, oral fluid vs. healthy oral fluid

## Abbreviations

PI: Plaque index
PPD: Probing pocket depth
BOP: Bleeding on probing
CAL: Clinical attachment loss
SRP: Scaling and root planning
PISF: Peri-Implant sulcus fluid
PICF: Peri-implant crevicular fluid
MMP: Matrix metalloproteinase
MMP-8, etc.: Matrix metalloproteinase-8
aMMP-8: Active form of matrix metalloproteinase-8
AAP: Asymptomatic apical periodontitis
(IL) IL-6, 8, etc.: Interleukins
(IL)-1*β*: Interleukin-1 beta
OPG: Osteoprotegerin
Dkk-1: Dickkopf-related protein 1
PTN: Periostin
TRAP-5: Tartrate-resistant acid phosphatase-5
ON: Osteonectin
TNF-*α*: Tumor necrosis factor-alpha
IFN-*γ*: Interferon gamma
TRACP-5b: Tartrate-resistant acid phosphatase serum type-5b
CTx: *C*-terminal cross-linked telopeptide of type I collagen
NTx: *N*-terminal cross-linked telopeptide of type I collagen
PMN: Polymorphonuclear leukocyte
*P. gingivalis:*: *Porphyromonas gingivalis*
*A. actinomycetemcomitans:*: *Aggregatibacter actinomycetemcomitans* (previously *Actinobacillus actinomycetemcomitans*)
*C. rectus:*: *Campylobacter rectus*
*F. nucleatum:*: *Fusobacterium nucleatum*
*P. intermedia:*: *Prevotella intermedia*
*T. forsythia:*: *Tannerella forsythia* (previously *Tannerella forsythensis*)
*T. denticola:*: *Treponema denticola*
PCR: polymerase chain reaction
GCF: Gingival crevicular fluid
IFMA: Immunofluorometric assay
ELISA: Enzyme-linked immunosorbent assay
TIMP-1: Tissue inhibitor of matrix metalloproteinase
ICTP: Pyridinoline cross-linked carboxy-terminal telopeptide of type I collagen
AgP: Aggressive periodontitis
CRS: Cumulative risk score
MIP-1*α*: Macrophage inflammatory protein
PGE2: Prostaglandin E2
PI: Peri-implantitis
CP: Chronic periodontitis
G: Gingivitis
GAgP: Generalized aggressive periodontitis
PoC: Point-of-care
POCID: Point-of-care immunoflow device
APD periodontal: Peri-implant degeneration
L/SDD: Low-dose doxycycline/sub-antimicrobial-dose doxycycline
AUC: Area under the curve
CRS: Cumulative risk score.

## References

[1] Kinane D. F. (2000). Regulators of tissue destruction and homeostasis as diagnostic aids in periodontology. *Periodontology 2000*.

[2] Nagase H., Visse R., Murphy G. (2006). Structure and function of matrix metalloproteinases and TIMPs. *Cardiovascular Research*.

[3] Sorsa T., Tjaderhane L., Konttinen Y. T. (2006). Matrix metalloproteinases: contribution to pathogenesis, diagnosis and treatment of periodontal inflammation. *Annals of Medicine*.

[4] Sorsa T., Tjaderhane L., Salo T. (2004). Matrix metalloproteinases (MMPs) in oral diseases. *Oral Diseases*.

[5] Bernasconi L., Ramenzoni L. L., Al-Majid A. (2015). Elevated matrix metalloproteinase levels in bronchi infected with periodontopathogenic bacteria. *PLoS One*.

[6] Sorsa T., Hernández M., Leppilahti J., Munjal S., Netuschil L., Mäntylä P. (2010). Detection of gingival crevicular fluid MMP-8 levels with different laboratory and chair-side methods. *Oral Diseases*.

[7] Sorsa T., Ulvi K., Nwhator S. (2016). Analysis of matrix metalloproteinases, especially MMP-8, in GCF, mouthrinse and saliva for monitoring periodontal diseases. *Periodontology 2000*.

[8] Sorsa T., Mäntylä P., Tervahartiala T., Pussinen P. J., Gamonal J., Hernandez M. (2011). MMP activation in diagnostics of periodontitis and systemic inflammation. *Journal of Clinical Periodontology*.

[9] Sorsa T., Uitto V. J., Suomalainen K., Vauhkonen M., Lindy S. (1988). Comparison of interstitial collagenases from human gingiva, sulcular fluid and polymorphonuclear leukocytes. *Journal of Periodontal Research*.

[10] Cox S. W., Eley B. M., Kiili M., Asikainen A., Tervahartiala T., Sorsa T. (2006). Collagen degradation by interleukin-1beta-stimulated gingival fibroblasts is accompanied by release and activation of multiple matrix metalloproteinases and cysteine proteinases. *Oral Diseases*.

[11] Gangbar S., Overall C. M., McCulloch C. A. G., Sodek J. (1990). Identification of polymorphonuclear leukocyte collagenase and gelatinase activities in mouthrinse samples: correlation with periodontal disease activity in adult and juvenile periodontitis. *Journal of Periodontal Research*.

[12] Mauramo M., Ramseier A. M., Mauramo E. (2017). Association of oral fluid MMP-8 with periodontitis in swiss adult subjects. *Oral Diseases*.

[13] Hernàndez M., Gamonal J., Tervahartiala T. (2010). Associations between matrix metalloproteinase-8 and 14 and myeloperoxidase in gingival crevicular fluid from subjects with progressive chronic periodontitis: a longitudinal study. *Journal of Periodontology*.

[14] Thierbach R., Maier K., Sorsa T., Mäntylä P. (2016). Peri-implant sulcus fluid (PISF) matrix metalloproteinase (MMP)-8 levels in peri-implantitis. *Journal of Clinical and Diagnostic Research*.

[15] Johnson N., Ebersole J. L., Kryscio R. J. (2016). Rapid assessment of oral salivary MMP-8 and periodontal disease using lateral flow immunoassay. *Oral Diseases*.

[16] Kinane D. F., Stathopoulou P. G., Papapanou P. N. (2017). Authors’ reply: predictive diagnostic tests in periodontal diseases. *Nature Reviews Disease Primers*.

[17] Sorsa T., Gieselmann D., Arweiler N. B., Hernández M. A. (2017). A quantitative point of care test for periodontal and dental peri implant diseases. *Nature Reviews Disease Primers*.

[18] Nwhator S. O., Ayanbadejo P. O., Umeizudike K. A. (2014). Clinical correlates of a lateral-flow immunoassay oral risk indicator. *Journal of Periodontology*.

[19] Yakob M., Kari K., Tervahartiala T. (2012). Associations of periodontal microorganisms with oral salivary proteins and MMP-8 in gingival crevicular fluid. *Journal of Clinical Periodontology*.

[20] Van Lint P., Libert C. (2006). Matrix metalloproteinase-8: cleavage can be decisive. *Cytokine & Growth Factor Reviews*.

[21] Ma J., Kitti U., Teronen O. (2000). Collagenases in different categories of peri-implant vertical bone loss. *Journal of Dental Research*.

[22] Kivelä-Rajamäki M. J., Maisi P., Srinivas R. (2003). Levels and molecular forms of MMP-7 (matrilysin-1) and MMP-8 (collagenase-2) in diseased human peri-implant sulcular fluid. *Journal of Periodontal Research*.

[23] Kivelä-Rajamäki M. J., Teronen O. P., Maisi P. (2003). Laminin-5 gamma2-chain and collagenase-2 (MMP-8) in human peri-implant sulcular fluid. *Clinical Oral Implants Research*.

[24] Mäntylä P., Stenman M., Kinane D. F. (2003). Gingival crevicular fluid collagenase-2 (MMP8) test stick for chair-side monitoring of periodontitis. *Journal of Periodontal Research*.

[25] Emingil G., Atilla G., Sorsa T., Luoto H., Kirilmaz L., Baylas H. (2004). The effect of adjunctive low dose doxycycline therapy on clinical parameters and GCF MMP-8 levels in chronic periodontitis. *Journal of Periodontology*.

[26] Pozo P., Valenzuela M. A., Melej C. (2005). Longitudinal analysis of metalloproteinases, tissue inhibitors of metalloproteinases and clinical parameters in GCF from periodontitis affected patients. *Journal of Periodontal Research*.

[27] Sorsa T., Golub L. M. (2005). Is the excessive inhibition of matrix metalloproteinases (MMPs) by potent synthetic MMP inhibitors (MMPIs) desirable in periodontitis and other inflammatory diseases? that is: ‘Leaky’ MMPIs vs excessively efficient drugs. Letter to editor. *Oral Diseases*.

[28] Mäntylä P., Stenman M., Kinane D. F. (2006). Monitoring periodontal disease status in smokers and nonsmokers using a gingival crevicular fluid matrix metalloproteinase-8-specific chair-side test. *Journal of Periodontal Research*.

[29] Miller C. S., King C. P., Langub M. C., Kryscio R. J., Thomas M. V. (2006). Oral salivary biomarkers of existing periodontal disease: a cross-sectional study. *Journal of the American Dental Association*.

[30] Xu L., Yu Z., Lee H. M. (2008). Characteristics of collagenase-2 from gingival crevicular fluid and peri-implant sulcular fluid in periodontitis and peri-implantitis patients: pilot study. *Acta Odontologica Scandinavica*.

[31] Giannobile W. V. (2008). Host-response therapeutics for periodontal diseases. *Journal of Periodontology*.

[32] Preshaw P. M. (2008). Host response modulation in periodontics. *Periodontol 2000*.

[33] Alfant B., Shaddox L. M., Tobler J., Magnusson I., Aukhil I., Walker C. (2008). Matrix metalloproteinase levels in children with aggressive periodontitis. *Journal of Periodontology*.

[34] Passoja A., Ylipalosaari M., Tervonen T., Raunio T., Knuuttila M. (2008). Matrix metalloproteinase-8 concentration in shallow crevices associated with the extent of periodontal disease. *Journal of Clinical Periodontology*.

[35] Ramseier C. A., Kinney J. S., Herr A. E. (2009). Identification of pathogen and host-response markers correlated with periodontal disease. *Journal of Periodontology*.

[36] Reinhardt R. A., Stoner J. A., Golub L. M. (2010). Association of GCF biomarkers during periodontal maintenance with subsequent progressive periodontitis. *Journal of Periodontology*.

[37] Gursoy U. K., Könönen E., Pradhan-Palikhe P. (2010). Oral salivary MMP-8, TIMP-1, and ICTP as markers of advanced periodontitis. *Journal of Clinical Periodontology*.

[38] Heikkinen A. M., Sorsa T., Pitkaniemi J. (2010). Smoking affects diagnostic oral salivary periodontal disease biomarker levels in adolescents. *Journal of Periodontology*.

[39] Marcaccini A. M., Meschiari C. A., Zuardi L. R. (2010). Gingival crevicular fluid levels of MMP-8, MMP-9, TIMP-2, and MPO decrease after periodontal therapy. *Journal of Clinical Periodontology*.

[40] Gursoy U. K., Kononen E., Pussinen P. J. (2011). Use of host-and bacteria-derived salivary markers in detection of periodontitis: a cumulative approach. *Disease Markers*.

[41] Özçaka O., Biçakci N., Pussinen P., Sorsa T., Köse T., Buduneli N. (2011). Smoking and matrix metalloproteinases, neutrophil elastase and myeloperoxidase in chronic periodontitis. *Oral Diseases*.

[42] Leppilahti J. M., Ahonen M. M., Hernández M. (2011). Oral rinse MMP-8 point-of-care immuno test identifies patients with strong periodontal inflammatory burden. *Oral Diseases*.

[43] Sexton W. M., Lin Y., Kryscio R. J., Dawson D. R., Ebersole J. L., Miller C. S. (2011). Salivary biomarkers of periodontal disease in response to treatment. *Journal of Clinical Periodontology*.

[44] Kraft-Neumärker M., Lorenz K., Koch R. (2012). Full-mouth profile of active MMP-8 in periodontitis patients. *Journal of Periodontal Research*.

[45] Arakawa H., Uehara J., Hara E. S. (2012). Matrix metalloproteinase-8 is the major potential collagenase in active peri-implantitis. *Journal of Prosthodontic Research*.

[46] Kushlinskii N. E., Solovykh E. A., Karaoglanova T. B. (2012). Matrix metalloproteinases and inflammatory cytokines in oral fluid of patients with chronic generalized periodontitis and various construction materials. *Bulletin of Experimental Biology and Medicine*.

[47] Konopka L., Pietrzak A., Brzezińska-Błaszczyk E. (2012). Effect of scaling and root planing on interleukin-1*β*, interleukin-8 and MMP-8 levels in gingival crevicular fluid from chronic periodontitis patients. *Journal of Periodontal Research*.

[48] Lowry O. H., Rosebrough N. J., Farr A. L., Randall R. J. (1951). Protein measurement with the Folin phenol reagent. *Journal of Biological Chemistry*.

[49] Ritzer J., Lühmann T., Rode C. (2017). Diagnosing peri-implant disease using the tongue as a 24/7 detector. *Nature Communications*.

[50] Yakob M., Meurman J. H., Sorsa T., Söder B. (2013). Treponema denticola associates with increased levels of MMP-8 and MMP-9 in gingival crevicular fluid. *Oral Diseases*.

[51] Irshad M., Scheres N., Anssari Moin D. (2013). Cytokine and matrix metalloproteinase expression in fibroblasts from peri-implantitis lesions in response to viable porphyromonas gingivalis. *Journal of Periodontal Research*.

[52] Rathnayake N., Akerman S., Klinge B. (2013). Salivary biomarkers of oral health: a cross sectional study. *Journal of Clinical Periodontology*.

[53] Gursoy U. K., Könönen E., Huumonen S., Tervahartiala T., Pussinen P. J., Suominen A. L. (2013). Salivary type I collagen degradation end-products and related matrix metalloproteinases in periodontitis. *Journal of Clinical Periodontology*.

[54] Gonçalves P. F., Huang H., McAninley S. (2013). Periodontal treatment reduces matrix metalloproteinase levels in localized aggressive periodontitis. *Journal of Periodontology*.

[55] Ebersole J. L., Schuster J. L., Stevens J. (2013). Patterns of salivary analytes provide diagnostic capacity for distinguishing chronic adult periodontitis from health. *Journal of Clinical Immunology*.

[56] Kinney J. S., Morelli T., Oh M. (2014). Crevicular fluid biomarkers and periodontal disease progression. *Journal of Periodontology*.

[57] Syndergaard B., Al-Sabbagh M., Kryscio R. J. (2014). Oral salivary biomarkers associated with gingivitis and response to therapy. *Journal of Periodontology*.

[58] Salminen A., Gursoy U. K., Paju S. (2014). Oral salivary biomarkers of bacterial burden, inflammatory response, and tissue destruction in periodontitis. *Journal of Clinical Periodontology*.

[59] Leppilahti J. M., Hernández-Ríos P. A., Gamonal J. A. (2014). Matrix metalloproteinases and myeloperoxidase in GCF provide site-specific diagnostic value for chronic periodontitis. *Journal of Clinical Periodontology*.

[60] Leppilahti J. M., Kallio A. M., Tervahartiala T., Sorsa T., Mäntylä P. (2014). Gingival crevicular fluid matrix metalloproteinase-8 levels predict treatment outcome among smokers with chronic periodontitis. *Journal of Periodontology*.

[61] Ebersole J. L., Nagarajan R., Akers D., Miller C. S. (2015). Targeted salivary biomarkers for discrimination of periodontal health and disease(s). *Frontiers in Cellular and Infection Microbiology*.

[62] Izadi Borujeni S., Mayer M., Eickholz P. (2015). Activated matrix metalloproteinase-8 in saliva as diagnostic test for periodontal disease? a case-control study. *Medical Microbiology and Immunology*.

[63] Leppilahti J. M., Sorsa T., Kallio M. A. (2015). The utility of gingival crevicular fluid matrix metalloproteinase-8 response patterns in prediction of site-level clinical treatment outcome. *Journal of Periodontology*.

[64] Heikkinen A. M., Nwhator S. O., Rathnayake N., Mäntylä P., Vatanen P., Sorsa T. (2016). Pilot study on oral health status as assessed by an active matrix metalloproteinase-8 chairside mouthrinse test in adolescents. *Journal of Periodontology*.

[65] Ramseier C. A., Eick S., Brönnimann C., Buser D., Brägger U., Salvi G. E. (2016). Host-derived biomarkers at teeth and implants in partially edentulous patients. A 10-year retrospective study. *Clinical Oral Implants Research*.

[66] Golub L. M., Elburki M. S., Walker C. (2016). Non-antibacterial tetracycline formulations: host-modulators in the treatment of periodontitis and relevant systemic diseases. *International Dental Journal*.

[67] Wang H. L., Garaicoa-Pazmino C., Collins A., Ong H-S., Chudri R., Giannobile W. V. (2016). Protein biomarkers and microbial profiles in peri-implantitis. *Clinical Oral Implants Research*.

[68] Janska E., Mohr B., Wahl G. (2016). Correlation between peri-implant sulcular fluid rate and expression of collagenase2 (MMP8). *Clinical Oral Investigations*.

[69] Baeza M., Garrido M., Hernández-Ríos P. (2016). Diagnostic accuracy for apical and chronic periodontitis biomarkers in gingival crevicular fluid: an exploratory study. *Journal of Clinical Periodontology*.

[70] Heikkinen A. M., Raivisto T., Kettunen K. (2017). Pilot study on the genetic background of an active matrix metalloproteinase-8 test in finnish adolescents. *Journal of Periodontology*.

[71] Noack B., Kipping T., Tervahartiala T., Sorsa T., Hoffmann T., Lorenz K. (2017). Association between serum and oral matrix metalloproteinase-8 levels and periodontal health status. *Journal of Periodontal Research*.

[72] Kumar Y., Jain V., Chauhan S., Bharati V., Koli D., Kumar M. (2017). Influence of different forms and materials (zirconia or titanium) of abutments in peri implant soft-tissue healing using matrix metalloproteinase-8: a randomized pilot study. *Journal of Prosthetic Dentistry*.

[73] Ziebolz D., Schmalz G., Gollasch D., Eickholz P., Rinke S. (2017). Microbiological and aMMP-8 findings depending on peri-implant disease in patients undergoing supportive implant therapy. *Diagnostic Microbiology and Infectious Disease*.

[74] Owen C. A., Hu Z., Lopez-Otin C., Shapiro S. D. (2004). Membrane-bound matrix metalloproteinase-8 on activated polymorphonuclear cells is a potent, tissue inhibitor of metalloproteinase-resistant collagenase and serpinase. *Journal of Immunology*.

[75] Uitto V. J., Suomalainen K., Sorsa T. (1990). Oral salivary collagenase: origin, characteristics and relationship to periodontal health. *Journal of Periodontal Research*.

[76] Lee W., Aitken S., Sodek J., McCulloch C. A. (1995). Evidence of a direct relationship between neutrophil collagenase activity and periodontal tissue destruction in vivo: role of active enzyme in human periodontitis. *Journal of Periodontal Research*.

[77] Kiili M., Cox S. W., Chen H. Y. (2002). Collagenase-2 (MMP-8) and collagenase-3 (MMP-13) in adult periodontitis: molecular forms and levels in gingival crevicular fluid and immunolocalisation in gingival tissue. *Journal of Clinical Periodontology*.

[78] Alassiri S., Parnanen P., Rathnayake N. (2018). Ability of quantitative, specific and sensitivity point-of-care/chair-side oral fluid immunotests for aMMP-8 to detect periodontal and per-implant diseases. *Disease Markers*.

[79] Sahrmann P., Betschart C., Wiedemeier D. B., Al-Majid A., Attin T., Schmidlin P. R. Treatment of peri-implant mucositis with a repeated chlorhexidine chipapplication during SPT–a randomized controlled clinical trial. *Clinical Oral Implants Research*.

[80] Mancini S., Romanelli R., Laschinger C. A., Overall C. M., Sodek J., McCulloch C. A. (1999). Assessment of a novel screening test for neutrophil collagenase activity in the diagnosis of periodontal diseases. *Journal of Periodontology*.

[81] Romanelli R., Mancini S., Laschinger C. (1999). Activation of neutrophil collagenase in periodontitis. *Infection and Immunity*.

[82] Teronen O., Konttinen Y. T., Lindqvist C. (1997). Human neutrophil collagenase MMP-8 in peri-implant sulcus fluid and its inhibition by clodronate. *Journal of Dental Research*.

[83] Sorsa T., Ding Y. L., Ingman T. (1995). Cellular source, activation and inhibition of dental plaque collagenase. *Journal of Clinical Periodontology*.

[84] Rathnayake N., Gieselmann D., Heikkinen A. M., Tervahartiala T., Sorsa T. (2017). Salivary diagnostics-point-of-care diagnostics of MMP-8 in dentistry and medicine. *Diagnostics*.

[85] Armitage G. C. (1999). Development of a classification system for periodontal diseases and conditions. *Annals of Periodontology*.

[86] Sorsa T., Ingman T., Suomalainen K. (1992). Identification of proteases from periodontopathogenic bacteria as activators of latent human neutrophil and fibroblast-type interstitial collagenases. *Infection and Immunity*.

[87] Lehtonen O. P., Grahn E. M., Stahlberg T. H., Laitinen L. A. (1984). Amount and avidity of salivary and serum antibodies against streptococcus mutans in 2 groups of human subjects with different dental-caries susceptibility. *Infection and Immunity*.

[88] Nieminen M. T., Listyarifah D., Hagström J. (2017). Treponema denticola chymotrypsin-like proteinase may contribute to orodigestive carcinogenesis through immunomodulation. *British Journal of Cancer*.

[89] Mombelli A., Lang N. P. (1998). The diagnosis and treatment of peri-implantitis. *Periodontol 2000*.

[90] Lang N. P., Wilson T. G., Corbet E. F. (2000). Biological complications with dental implants: their prevention, diagnosis and treatment. *Clinical Oral Implants Research*.

[91] Berglundh T., Persson L., Klinge B. (2002). A systematic review of the incidence of biological and technical complications in implant dentistry reported in prospective longitudinal studies of at least 5 years. *Journal of Clinical Periodontology*.

[92] de Morais E. F., Pinheiro J. C., Leite R. B., Santos P. P. A., Barboza C. A. G., Freitas R. A. (2017). Matrix metalloproteinase-8 levels in periodontal disease patients: a systematic review. *Journal of Periodontal Research*.

[93] Jentsch H. F., Buchmann A., Friedrich A., Eick S. (2016). Nonsurgical therapy of chronic periodontitis with adjunctive systemic azithromycin or amoxicillin/metronidazole. *Clinical Oral Investigations*.

